# Rational Design of *Aspergillus awamori* Glucoamylase for Activity Improvement

**DOI:** 10.3390/biotech15040074

**Published:** 2026-09-02

**Authors:** Anna Dotsenko, Nikita Eroshenko, Ekaterina Rubtsova, Aleksandra Rozhkova, Ivan Zorov, Arkady Sinitsyn

**Affiliations:** 1 Federal Research Centre ”Fundamentals of Biotechnology” of the Russian Academy of Sciences, Moscow 119071, Russia; nikita.eroshenko@chemistry.msu.ru (N.E.); katefedo@yandex.ru (E.R.); a.rojkova@fbras.ru (A.R.); inzorov@mail.ru (I.Z.); apsinitsyn@enzyme.chem.msu.ru (A.S.); 2 Department of Chemistry, M.V. Lomonosov Moscow State University, Moscow 119991, Russia

**Keywords:** glucoamylase, rational design, activity, starch, substrate binding, homodimer, glycosylation, *Aspergillus*

## Abstract

Glucoamylases are highly demanded by the industry for starch saccharification, which necessitates an increase in enzyme activity. To improve the activity of *Aspergillus awamori* VKPM F-1262 glucoamylase N181Q (AGAM), amino acid substitutions were made in the region of substrate binding. In the case of the variant D237G, there was a 1.3–1.4-fold increase in activity towards soluble starch for the individual enzyme and crude enzyme solution after cultivation in flasks. For the variants G57A and C319T, there was also a 1.1–1.4-fold increase in enzyme activity for the crude flask solution, while the increase in individual enzyme activity was minor. AGAM showed an increase in apparent molecular weight consistent with dimerization in the presence of soluble starch. A high T optimum of 70 °C was observed for AGAM and the variants in the hydrolysis of soluble starch. In further protein engineering, a high T optimum for hydrolysis, which may coincide with dimerization, may be considered as a hypothesis for the selection of thermostable glucoamylases, while an area of binding of oligo- and polysaccharides may be a site for mutagenesis.

## 1. Introduction

Glucoamylases (GAMs) are enzymes that primarily hydrolyze α-1,4 and partially α-1,6 glycosidic bonds at the non-reducing ends of naturally occurring polysaccharide starch and related oligo- and polysaccharides [1]. GAMs are classified under EC 3.2.1.3 and are highly demanded by the industry for starch saccharification. They account for 25% of the enzymes used in industry, along with other starch-hydrolyzing enzymes such as α-amylases and debranching enzymes [2].

An improvement in the enzymatic activity of GAMs is needed to reduce the cost of starch saccharification. Various attempts have been made to increase the activity of GAMs towards starch through rational design. A 1.2-fold increase in *Aspergillus awamori* GAM activity was achieved through amino acid (AA) substitutions G137A/N20C/A27C and G137A/S436P [3,4]. The N20C-A27C disulfide bond was engineered close to W120, which was critical for substrate binding [4]. Two other substitutions, G137A and S436P, reduced flexibility of the α-helix [5] and the C-terminus of the catalytic domain [6], respectively. Another substitution near the active center of *A. awamori* GAM, A393P, also increased activity by 1.2 times [6].

Directed evolution led to an improvement similar to that achieved through rational design. Single substitutions V301D and T390A resulted in a 1.2-fold and 1.4-fold increase in the activity of *A. awamori* GAM, respectively, with different structural bases for this improvement. V301D stabilized a loop connecting two α-helices, while T390A reduced the local tension of an α-helix linked to a loop containing the catalytic residue E400 [7]. A substitution Q113M, obtained through saturation mutagenesis in *A. fumigatus* GAM, increased the enzyme’s activity by 1.5 times [8].

A different approach, a modification of the domain structure, has also led to an increase in the activity of GAMs. GAMs typically consist of a catalytic domain (CD), a linker, and a starch-binding domain (SBD). The CD contains two catalytic residues of glutamic acid in the active center, providing exo-activity on poly- and oligosaccharide substrates. The glycosylated linker and the SBD are crucial for hydrolysis of raw starch because they bind to starch granules, disrupting their packed structure and releasing single polysaccharide chains that are then acted on by the CD [9]. Covalent binding of the linker to CD through two disulfide bonds, S132C-Y492C/L548C-A562C, in *Talaromyces leycettanus* JCM12802 GAM resulted in a 1.6-fold increase in activity [10]. However, replacement of the linker in *A. niger* GAM with shorter natural linkers or a constructed proline-rich linker did not affect the enzyme’s activity towards starch but did decrease its stability [9]. Additionally, the glycosylated linker in *A. niger* GAM was reported to form a compact, folded area of the spatial structure rather than an elongated, flexible strand. This close position of the CD and SBD was thought to enable dimerization of this GAM during starch hydrolysis [1].

As far as heterologous hosts are used for industrial-scale production of enzymes, there is a demand for both increased activity of individual enzymes and increased production of crude enzyme solutions in order to reduce costs. For example, substitutions G57A and N182Q have increased the production of *A. awamori* GAM in *Saccharomyces cerevisiae* by 1.6 and 1.4 times, respectively, without affecting the activity of the individual enzyme [5,11]. The substitution G57A was located in the inner α-helix of CD and was supposed to promote faster biosynthesis and improved folding of the enzyme, as well as higher intracellular stability during production [5]. The substitution N182Q eliminated the theoretical site of *N*-linked glycosylation, which carried no glycan and was located in the active center [11]. In contrast, creation of a new site of *N*-linked glycosylation in the CD of *A. awamori* GAM through substitution D238N was reported to increase enzyme production in *S. cerevisiae* by 1.5–2 times without any effect on its activity and thermostability [12].

Therefore, improving the activity of Aspergillus GAMs is a challenging task due to the reported increase of only 1.2–1.6 times. Additionally, the spatial structure of full-length GAMs containing CD and SBD remains an open question. Dimerization during starch hydrolysis was hypothesized for *A. niger* GAM, but it has not been investigated [1]. The goals of the present study were (1) to rationally design *A. awamori* VKPM F-1262 GAM to improve its activity; (2) to distinguish the effects on the activity of the individual enzyme and the activity in the crude culture solution obtained from fermentation; and (3) to investigate the spatial structure of the enzyme. Rational design focused on the substrate-binding area in the CD in order to affect the enzyme’s catalytic function. The number of AA substitutions was further restricted by analysis of multiple sequence alignment and ΔΔG calculations to eliminate substitutions that disrupt the CD’s spatial structure. A heterologous host, *Penicillium verruculosum* B1-537, was used for enzyme production, and the activity in the crude culture solution produced by the transformants was measured. The effect of AA substitutions on the activity and thermostability of the purified enzyme was investigated. In the case of the spatial structure, glycosylation, disulfide bonds, and mobility in polyacrylamide gels under native and denaturing conditions, and in the presence of soluble starch, were investigated.

## 2. Materials and Methods

### 2.1. Rational Design

*Aspergillus awamori* VKPM F-1262 GAM with the substitution N181Q (AGAM) was the object of this study. The substitution N181Q was previously performed in *A. awamori* VKPM F-1262 GAM [13] based on the positive effect reported for the same substitution in GAM produced by a different strain of *A. awamori* [11]. The tertiary structure of the catalytic domain of AGAM was modeled using SwissModel [14]. An oligosaccharide with eight glucose residues, Glc-1,4-α-8, was modeled with CHARMM-GUI Glycan Modeler [15] and docked into the active center of AGAM using AutoDock Vina 1.2.1 [16] within AMDock 1.5.2 [17].

ΔΔG calculations were performed using Maestro [18,19], RosettaDesign [20], FoldX [21], ENCoM [22], and SDM [23,24]. ConSurf and multiple sequence alignment were used to analyze the conservation of AA positions [25,26]. The agreement of the mentioned predictors was considered in order to improve predictions [27].

### 2.2. Site-Directed Mutagenesis and P. verruculosum Transformation

Target mutations were introduced into the AGAM gene [13] using the QuikChange method [28]. Site-directed mutagenesis was performed using oligonucleotide primers listed in Table S1, as described elsewhere [13]. The host strain *P. verruculosum* B1-537 (niaD^−^) (VKM F-3972D, All-Russian Collection of Microorganisms, Pushchino, Russia) was transformed with plasmids using a method described elsewhere [13]. A single transformation was carried out for each plasmid containing a mutated or original AGAM gene. The total number of protoplasts from the recipient culture was aliquoted into several tubes and used for transformation with plasmids. One tube was used for each transformation with a particular plasmid.

### 2.3. Production in P. verruculosum

For the primary screening, transformants were cultured in 1.5 mL of liquid medium for 6 days at 32 °C with constant shaking at 180 rpm in plates. The medium composition was (g/L): KH_2_PO_4_—10.0; K_2_HPO_4_—1.0; (NH_4_)_2_SO_4_—5.0; CaCl_2_·2H_2_O—0.3; MgSO_4_·7H_2_O—0.3; cellulose—40.0; yeast extract—10.0; wheat bran—10.0. Transformants with the highest glucoamylase activity towards soluble starch were selected for further cultivation in Erlenmeyer flasks. For this purpose, Erlenmeyer 750 mL flasks containing 100 mL of the same medium were used. The conditions of cultivation and composition of the medium remained the same. After centrifugation, crude enzyme solution was collected and used for enzyme purification.

### 2.4. Purification

AGAM and the variants were purified using anion-exchange chromatography, as described elsewhere [29]. Briefly, crude enzyme was precipitated using (NH_4_)_2_SO_4_ to remove medium components, then desalted and purified through anion-exchange chromatography in a NaCl gradient. The buffer was changed to 0.05 M Na-acetate buffer, pH 4.7, using membrane columns. Protein concentration was determined spectrophotometrically at 280 nm using an extinction coefficient calculated by ProtParam, https://web.expasy.org/protparam/ (accessed on 27 March 2026) [30].

### 2.5. Characterization of Catalytic and Biochemical Properties

The hydrolysis of 0.8% soluble potato starch (Panreac Quimica SLU, Barcelona, Spain) was carried out for 10 min at 30 °C and pH 4.7 (0.05 M Na-acetate buffer) and stopped by heating at 100 °C for 30 min. The concentration of glucose was then measured using a glucose oxidase method (glucose measuring kit, Photoglucose, Impact, Moscow, Russia). Enzyme activity was calculated from the glucose concentration, and one unit of activity corresponded to the amount of enzyme that released 1 μmol of glucose per minute.

Enzyme activity towards soluble starch was tested over a pH range of 1.5–6.0 (0.05 M Britton-Robinson buffer) at 70 °C. Activity was also measured over a T range of 30–90 °C at a fixed pH of 4.7.

Catalytic parameters were determined for the hydrolysis reaction of soluble starch, with substrate concentrations ranging from 0.25 to 18 g/L and pH 4.7 at 30 °C. Parameters were calculated using a hyperbolic fit in OriginPro 8.5.

All assays for the characterization of these properties were performed in triplicate. Data are presented as mean ± standard deviation.

### 2.6. Mass Spectrometry

The AA sequence of AGAM and the variants was confirmed by MALDI-TOF/TOF mass spectrometry (UltrafleXtreme mass spectrometer, Bruker Daltonik GmbH, Bremen, Germany), as described for other glycoside hydrolases elsewhere [31,32]. The sites of *N*-linked glycosylation and the structure of glycans were assigned using Glycomod, https://web.expasy.org/glycomod/ (accessed on 8 June 2026) [33].

### 2.7. Characterization of Glycosylation

The degree of total glycosylation, consisting of *N*- and *O*-linked glycosylation, was measured using the phenol-sulfuric method of Dubois [34]. D-mannose (Panreac, Quimica S.A.U., Barcelona, Spain) was used as a standard, with concentrations ranging from 5 to 50 μg/mL. The calculation formula used was C (μg/mL) = OD/0.0133, where OD represents optical density in optical units. The absorbance was measured in comparison with two blanks: the phenol-sulfuric reagent and the enzyme solution. Measurements were performed in triplicate, and data were presented as mean ± standard deviation.

The types of *N*-linked glycans were tested using treatments with Endo F1, Endo F2, and Endo F3 (Native Protein Deglycosylation Kit, Sigma-Aldrich, St. Louis, MO, USA), as well as PNGase F from *Elizabethkingia meningoseptica* (Sigma-Aldrich). The sites and structure of the *N*-linked glycans were putatively assigned by MALDI-TOF mass matching with Glycomod (Section 2.6), supported by glycosidase sensitivity.

### 2.8. Detection of Sulfhydryl Groups

The presence of free sulfhydryl groups due to cysteine residues not involved in disulfide bridges was tested using the modified method of Ellman [35], as described elsewhere [36]. To detect free sulfhydryl groups in the native and denatured states, aliquots of the protein were stored in 0.05 M Na-acetate buffer (pH 4.7) for 5 min at room temperature, heated at 100 °C for 5 min in the same buffer, or treated with guanidine hydrochloride (Dia-M, Moscow, Russia) at 100 °C for 5 min for denaturation. Measurements were performed in triplicate, with data presented as mean ± standard deviation.

### 2.9. Differential Scanning Fluorimetry

Melting temperature (*T_m_*) was determined using differential scanning fluorimetry on a Varian Cary Eclipse fluorescence spectrophotometer (Agilent Technologies, Palo Alto, CA, USA) with a Cary single-cell Peltier accessory (Agilent Technologies, USA) and a fluorescent marker, chromotrope 2B (2-(4-nitrophenylazo) chromotropic acid disodium salt). The buffer used was the same as that used for activity measurements (0.05 M Na-acetate buffer, pH 4.7). The heating rate was 1 °C/min, and the excitation and emission wavelengths were set at 300 and 600 nm, respectively, as previously described [36].

### 2.10. Electrophoresis in Polyacrylamide Gel

Sodium dodecyl sulfate-polyacrylamide gel electrophoresis (SDS-PAGE) was performed using different concentrations of separation gel, ranging from 4.0 to 8.0%. The protein samples were heated at 100 °C in the presence of bromophenol blue (BPB, 0.05%), sodium dodecyl sulfate (SDS, 1%), and 2-mercaptoethanol (2-ME, 5%) before being applied to the gel. Protein bands were then used for MALDI-TOF/TOF mass spectrometry (Section 2.6).

Native-Blue electrophoresis was conducted without the addition of SDS or 2-ME, and the protein samples were not preheated. Under Native-Clean electrophoresis conditions, BPB was also excluded. As a modification of Native-Blue electrophoresis, 0.015 M urea was added to the polyacrylamide gel in order to perform electrophoresis while blocking hydrophobic interactions. Gels obtained after electrophoresis were stained using Coomassie blue G-250 (Helicon, Moscow, Russia).

### 2.11. Zymography

To detect enzyme activity for the protein band in the polyacrylamide gel, two strategies were used for electrophoresis. In the first strategy, Native-Clean electrophoresis was performed as described in Section 2.10. Afterward, the gel was washed with 0.05 M Na-acetate buffer (pH 4.7) for 30 min, incubated with a 0.8% solution of starch in 0.05 M Na-acetate buffer (pH 4.7) at room temperature for 60 min, washed again with buffer solution, and incubated at 30 °C for 30 min. Finally, the gel was colored with I_2_ solution. In the second strategy, the gel was prepared with 0.8% soluble starch and used for Native-Clean electrophoresis. It was verified that the solution of starch did not affect the pH of the mixture of acrylamide, bisacrylamide, and Tris buffer (pH 8.8) used for gel preparation. The concentration of soluble starch (0.8%) was the same as in the activity assay and saturating, according to the *K_m_* value (Section 2.5). After electrophoresis, the gel was washed with 0.05 M Na-acetate buffer (pH 4.7) for 30 min and incubated at 30 °C for 30 min. The resulting gel was then colored with I_2_ solution. In both strategies, white bands were observed in the blue-stained gel. Each strategy was run in duplicate, one of which was used for activity staining and the other for Coomassie blue G-250 staining (Helicon).

### 2.12. Molecular Dynamics

A full-length model of AGAM with a catalytic domain, a linker, and a starch-binding domain was generated using AlphaFold 2.0 [37]. A model of AGAM with *N*- and *O*-linked glycans was generated using Glycan Reader and Modeler [15,38,39], available through the CHARMM-GUI web service [40,41]. The structure of *N*-linked glycans was (Man)_8_(GlcNAc)_2_ at glycosylation sites N170 and N394, according to MALDI-TOF analysis (Section 2.6). *O*-linked glycans were (Man)_1_ for serine and threonine residues in the sequence S442–T463, according to the tertiary structure of *A. awamori* GAM (PDB ID 1GAI), and (Man)_2_ in the sequence T474–S506, according to reported studies [9,42]. A water box was rectangular, with an edge distance of 20 Å, 0.1 mol/L concentrations of sodium and chloride ions, and a neutral charge. Molecular dynamics (MD) simulations of a monomer of AGAM were performed at 30 °C for 75 ns in a single trajectory. The parameters for the input files for MD simulations were as described elsewhere [29].

The structure of the monomer, obtained after 75 ns of MD simulations at 30 °C, was aligned with the structure of *Hormoconis resinae* GAM (PDB/PISA ID 6FHW annotated as a biological assembly) [43] to form a homodimer. This homodimer was then equilibrated for 10 ns at 30 °C and simulated for another 75 ns at 100 °C in a single trajectory. The input file parameters were the same as those used in the MD simulation of the monomer. The simulations were performed using VMD 1.9.4 and NAMD 2.13 [44,45].

## 3. Results

### 3.1. Rational Design of AGAM

Several fungi of the *Aspergillus* genus, including different strains of *A. awamori*, are capable of producing GAMs. It was reported that GAMs produced by various strains of *A. awamori* and *A. niger* have an identical AA sequence [46]. However, these GAMs were only 94% identical to the AA sequence of the GAM from *A. awamori* var. *X100* [47]. These GAMs differ in their enzymatic properties due to differences in their AA sequences. Therefore, here and further, the strain will be specified or indicated if it is not reported (n.r.). In particular, *A. awamori* VKPM F-1262 GAM with the substitution N181Q was used in this study (AGAM) [13]. The substitution N181Q in *A. awamori* VKPM F-1262 GAM was previously performed [13] based on the positive effect reported for an equivalent substitution in *A. awamori* (strain n.r.) GAM [11].

**Figure 1:**
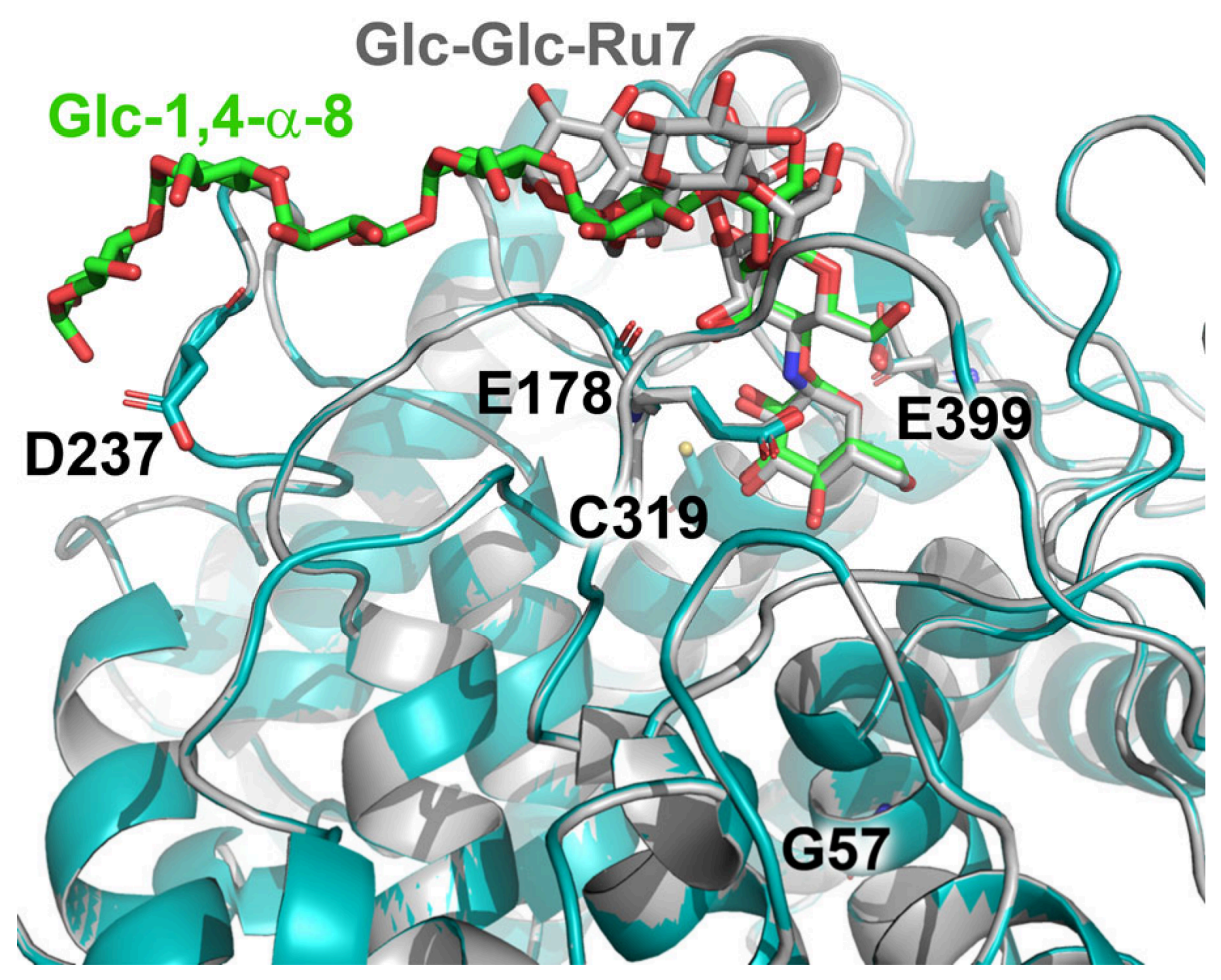
An alignment of the tertiary structure of the catalytic domain of *A. awamori* GAM (PDB ID 1GAI, shown in gray) and the modeled catalytic domain of AGAM (shown in cyan), which was obtained using PDB ID 1GAI as a template. The oligosaccharide Glc-1,4-α-8 (shown as green sticks) was docked into the active center of AGAM. The substrate analog Glc-Glc-Ru7 (shown as gray sticks) was extracted from PDB ID 1GAI. Red and blue colors correspond to O and N atoms, respectively.

The AA chain of AGAM contains a catalytic domain (CD) at the N-terminus of the sequence, a linker region, and a starch-binding domain (SBD) at the C-terminus. The CD of AGAM has 96% sequence identity with the CD of *A. awamori* (strain n.r.) GAM (PDB ID 1GAI and UniProt ID P69327), which is one of the most reported and engineered GAMs. Due to the high level of similarity, the tertiary structure 1GAI was used as a template for modeling the CD of AGAM (Figure 1).

In a rational design for improving AGAM activity, it was hypothesized that AA substitutions in the area of substrate binding in the CD could affect the catalytic function of the enzyme. To investigate this area, a molecule of oligosaccharide Glc-1,4-α-8 was docked into the active center of AGAM near the catalytic residues E178 and E399. The model with the lowest binding energy (−8.3 kcal/mol) was selected (Figure 1). The position of the terminal monosaccharide units in the docked Glc-1,4-α-8 matched the positions of equivalent units in the substrate analog Glc-Glc-Ru7 in the crystal structure of *A. awamori* (strain n.r.) GAM (PDB ID 1GAI), confirming the accuracy of docking. Additionally, “tail” parts of Glc-1,4-α-8 and Glc-Glc-Ru7 had similar positions in the general binding direction (Figure 1).

An area within 10 Å of atom O4 of the docked oligosaccharide Glc-1,4-α-8 and atoms CD of E178 and E399 was considered to be the area for potential oligosaccharide binding in AGAM. The AA residues in this area were therefore considered as possible sites for rational design. For these residues, the frequency of AA substitutions was counted in a multiple sequence alignment of AGAM with 150 homologous proteins with 35–95% sequence identity. Fourteen AA substitutions were found to be more frequent in the alignment compared to the amino acids present in the AGAM sequence at the same positions (Table 1).

Several services for ΔΔG calculation provided different predictions for AA substitutions (Table 1). While the overall accuracy of these predictors is reported to be low, they have been shown to have good specificity for predicting destabilizing substitutions [51]. In the rational design of AGAM for activity improvement, ΔΔG calculations were not used to improve enzyme thermostability, but rather to identify and eliminate substitutions that were predicted to be destabilizing and potentially disrupt the enzyme’s spatial structure. Most of the predictors applied suggested destabilization for 10 AA substitutions. However, for four AA substitutions—G57A, D237G, C319T, and E408P—stabilization was predicted (Table 1).

Of the substitutions G57A, D237G, C319T, and E408P, two were reported to affect the properties of GAMs. The substitution G57A improved the production of *A. awamori* (strain n.r.) GAM in *S. cerevisiae* C468 and had no effect on the activity or thermostability of the individual enzyme [5]. The substitution E408P in *A. awamori* (strain n.r.) GAM also had no effect on enzyme activity at 50 °C but did decrease thermostability [3]. The substitution C320A in *A. awamori* (strain n.r.) GAM, which was equal to C319A in AGAM due to differences in AA sequences, caused a slight change in activity and thermostability but decreased enzyme production in *S. cerevisiae* C468 [47,50] (Table 2). This latter finding was similar to, but not the same as, the substitution C319T in AGAM. The substitutions D237G and C319T have not been reported to our best knowledge. Therefore, they were chosen for investigation. The substitution G57A was also selected to test its influence on the production of AGAM in *P. verruculosum*. Another substitution, C319A, which has been reported to have a modest effect on the activity of *A. awamori* (strain n.r.) GAM [47,50] was chosen as an internal standard for evaluating the substitution C319T in AGAM.

### 3.2. Screening of Transformants Producing AGAM and Variants

A transformation of *P. verruculosum* B-537 resulted in 40–44 transformants for AGAM and the variants with AA substitutions. These transformants were cultivated on plates (Section 2.3), and the enzyme activity towards soluble starch was measured in the cultures after 6 days. In the case of AGAM, 44 transformants obtained differed in enzyme activity when normalized to total protein content. The highest activity was 12.5 ± 0.8 U/mg, while the lowest activity was below the detection level (Figure S1). Heterologous enzyme production in fungal hosts has been reported to depend on transformation efficiency and integration of heterologous genes into the genome. Therefore, it is necessary to screen for transformants that produce the same enzyme but in varying amounts [52]. Similar differences in the level of crude culture production, with only a few highly productive transformants from a screening set, were reported for heterologous production in *A. vadensis* [52] and *Pichia pastoris* [53].

In the case of the variants, a similar diversity of transformants in activity was obtained (Figure S1). The highest values of activity were 17.7 ± 1.1, 17.2 ± 1.1, 14.8 ± 1.0, and 12.4 ± 0.8 U/mg for the variants G57A, D237G, C319T, and C319A, respectively.

Three transformants demonstrating the highest enzyme activity after cultivation on plates were selected for each AGAM variant for further cultivation in flasks. In the case of AGAM and the variants, the three transformants cultivated in flasks differed in activity similarly to cultivation on plates. The highest activity values were 33.2 ± 2.7, 32.4 ± 2.1, 27.7 ± 2.7, and 21.4 ± 2.4 U/mg for the variants G57A, D237G, C319T, and C319A, respectively, while 24.2 ± 2.0 U/mg was obtained for AGAM (Figure S2). For the variants G57A and D237G, the activity was increased by 1.4 and 1.3 times, respectively, compared to AGAM.

As mentioned above, two substitutions in AGAM, D237G and C319T, are investigated in the present study. Two other substitutions, G57A and C319A, were previously reported. The substitution G57A was reported to improve production of *A. awamori* (strain n.r.) GAM in *S. cerevisiae* C468 without affecting the activity of the individual enzyme [5]. The substitution C320A in *A. awamori* (strain n.r.) GAM, equal to C319A in AGAM due to differences in AA sequences, was reported to cause a slight change in activity and thermostability, as well as decrease enzyme production in *S. cerevisiae* C468 [50]. To distinguish between the effects of AA substitutions on enzyme production in the host strain and individual enzyme activity, AGAM and the variants were purified using chromatography, and their properties were tested.

### 3.3. Catalytic and Biochemical Properties of AGAM and Variants

Anion-exchange chromatography was used to obtain purified enzymes (Figure S3). The activity of individual enzymes was 1.2–1.5 times higher than the activity in crude cultures of the best transformants (Table 3). This low fold increase in activity after purification was consistent with the high content of AGAM and the variants in crude cultures, where most of the produced protein was the target enzyme (Figure S3a,b for crude cultures and individual enzymes, respectively).

In the set of variants, only the variant D237G showed a 1.3-fold increase in activity when compared to AGAM. This substitution also led to a 1.3-fold increase in *k_cat_* and a 1.2-fold increase in *K_m_* in the starch hydrolysis reaction. The 1.3-fold improvement in activity was modest, but it was similar to the 1.2–1.5-fold improvements reported for Aspergillus GAMs through rational design and directed evolution [3,4,5,6,7,8], as well as the 1.6-fold improvement obtained by changing the domain structure [10], as mentioned in the introduction.

For other variants, changes in activity, *k_cat_*, and *K_m_* were all less than 1.15-fold. All variants had similar catalytic efficacies (*k_cat_*/*K_m_*) to AGAM (Table 3).

All variants and AGAM showed the highest activity at 70 °C, similar to the data reported for *A. niger* (strain n.r.) GAM [54]. Additionally, the variants and AGAM were active over a wide range of pH values, with more than 50% activity in the range of 1.5–5.5, more than 90% activity in the range of 2.5–4.5, and nearly 100% activity in the range of 3.0–4.0. Similar broad pH profiles of activity have been reported for GAMs produced by *A. niger* (strain n.r.) [55], *A. awamori* (strain n.r.) [56], *A. awamori* nakazawa (MTCC 6652) [57], *A. flavus* (strain n.r.) [2], and *A. niger* MTCC478 [58].

The variants D237G and C319A were similar to AGAM in *T_m_*, while the variants G57A and C319T were less thermostable (Table 4). Although the variants G57A and C319T had enzyme activity similar to that of AGAM, their lower thermal stability made them less suitable for industrial use compared to AGAM. In industrial starch hydrolysis processes with high starch concentrations, the substrate can protect enzymes from thermal inactivation [59], so a decrease in *T_m_* of −0.6 °C could be expected to have only a minor effect on starch hydrolysis efficacy.

In the rational design of AGAM, four AA substitutions were chosen based on their high frequencies in multiple sequence alignment. However, no association between these high frequencies and changes in *T_m_* was found (Table 4). Nevertheless, all variants were successfully produced and maintained enzyme activity. This confirms the usefulness of multiple sequence alignment in the search for enzymatically active variants.

In the predictions of ΔΔG, an association between the predicted values and the change in *T_m_* was not observed. Neutral ΔΔG values fell within the range of −0.5 to 0.5 kcal/mol or REU [51]. Destabilization was assigned with highly positive ΔΔG values by Maestro [18,19], RosettaDesign [20], and FoldX [21], while highly negative ΔΔG values were assigned by SDM [23,24]. In the ΔΔS prediction, positive values indicate higher molecular flexibility [22]. Among the tools used, FoldX and SDM correctly predicted two of four substitutions, while the predictions of Maestro and ENCoM were incorrect. The predictions of Rosetta were correct for the substitution C319T (Table 4). Although there were a small number of substitutions made, the data obtained were in accordance with the low accuracy reported for the predictors [51]. However, the goal of ΔΔG and ΔΔS calculations in the rational design of AGAM was to eliminate destabilizing substitutions. In experimental testing, none of the substitutions led to a considerable destabilization, with changes in *T_m_* ranging from −0.6 to 0.2 °C.

**Figure 2:**
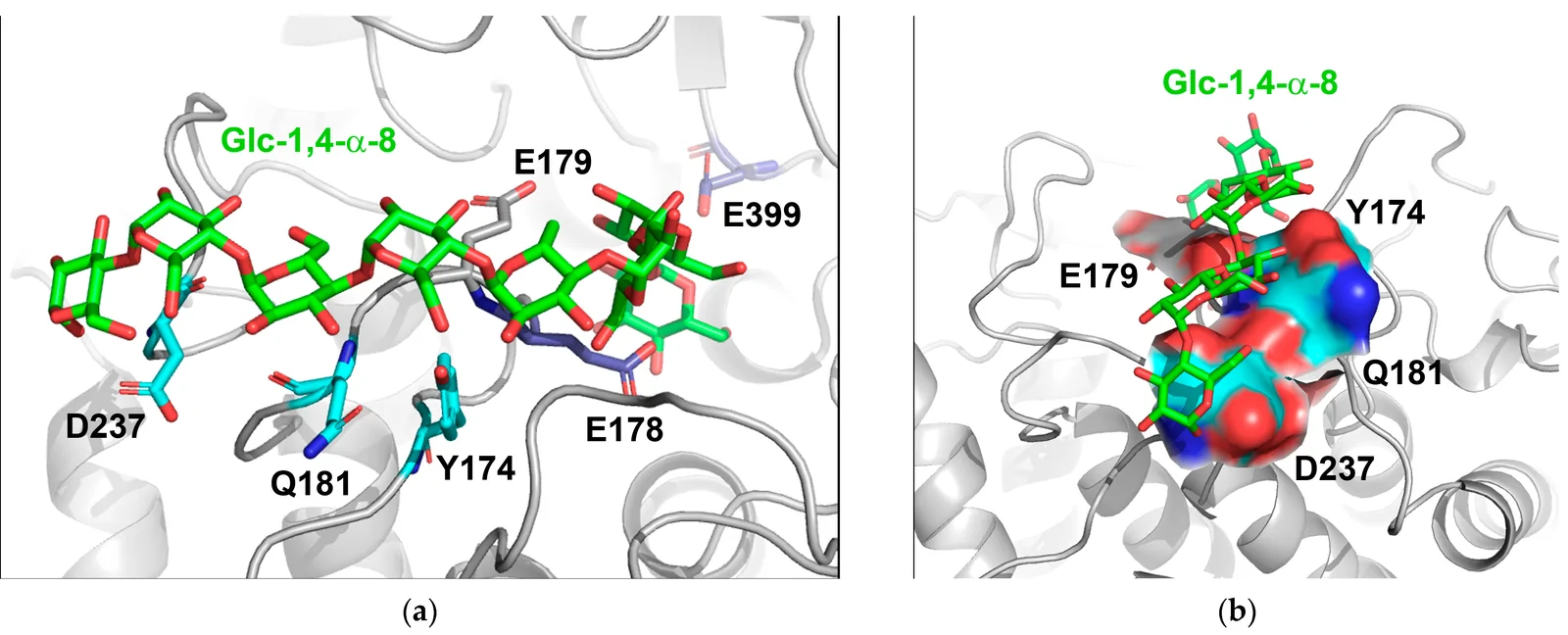
(**a**) Docking of Glc-1,4-α-8 (shown in green sticks) in the active center of AGAM. The residues Y174, Q181, and D237 are shown as cyan sticks. The catalytic residues E178 and E399 are shown as dark blue sticks. The residue E179 at the +1 subsite is shown as gray sticks. (**b**) The groove for substrate binding is formed by Y174, E179, Q181, and D237. Red and blue colors correspond to O and N atoms, respectively.

Among the substitutions performed, D237G differed in its location on the surface of the catalytic domain, being close to the docked oligosaccharide. Other substitutions, G57A, C319T, and C319A, were buried within the catalytic domain. Additionally, D237G was located near other residues known to affect glucoamylase activity, specifically E179, Q181, and Y174 (Figure 2a). These residues in AGAM, E179, Q181, and Y174, correspond to E180, N182, and Y175 in *A. awamori* and *A. niger* GAMs, with the difference in the position numbers resulting from the differences in AA sequences (Table 5).

The residues E180, N182, and Y175 in *A. awamori* and *A. niger* GAMs have been reported to induce a conformational change in the bound substrate at the +1 subsite [9,60], affect enzyme activity [11], and affect catalytic parameters [48,49], respectively (Table 5). In the spatial structure of AGAM, equal residues E179, Q181, and Y174 formed a compact area near the active center (Figure 2a). Together with residues E179, Q181, and Y174, the residue D237 formed an extended groove on the enzyme surface (Figure 2b).

Based on the increased catalytic parameters observed for the variant D237G, it can be inferred that this residue plays a role in substrate binding. Catalytic parameters are part of the Michaelis-Menten equation, which approximates kinetics for most enzymes, including those that hydrolyze starch [61]:

$$v=\;\frac{k_{cat}{\left[E\right]}_{0}\left[S\right]}{K_{m}+\left[S\right]}.$$

The simplest model that corresponds to the Michaelis-Menten equation is a two-step process, where the formation of an enzyme-substrate complex is followed by product release:

$$E+S\begin{array}{c} k_{1}\\ ⇄\\ \;k_{-1} \end{array}ES\;\begin{array}{c} k_{2}\\ \to \\ \; \end{array}E+P.$$

In this case, the catalytic parameters in the Michaelis-Menten equation are $k_{cat}=\;k_{2}$ and $K_{m}=\;\frac{k_{-1}+k_{2}}{k_{1}}$. For GAMs, the rate of reaction was proposed to be mainly affected by the hydrolysis step, when the glycosidic bond in the substrate is broken [9], and the step of product dissociation from the active center [62,63]. An increase in *k_cat_* and *K_m_* for the substitution D237G can be explained by a faster dissociation of the reaction product, oligo- or polysaccharide, from the active center. In the mentioned model of the reaction, the faster dissociation could increase the *k*_2_ value and therefore both *k_cat_* and *K_m_.*

### 3.4. Disulfide Bonds and Glycosylation of AGAM and Variants

The theoretical weight of the protein chain of AGAM was 65.9 kDa, and a molecular weight of 140 kDa was observed in SDS-PAGE (Figure S3c). This difference in molecular weight was similar to that reported for GAMs from *A. niger* (strain n.r.) and *A. niger* N402, but differed from that of GAMs from *A. niger* NIBGE-1, *A. awamori* (strain n.r.), and *A. niveus* WFCC604 (Table 6).

In order to investigate the reason for this difference, several characteristics of AGAM and the variants were tested. These included free thiol groups, the total degree of glycosylation, and the type and structure of *N*-glycosylation.

The AA sequence of AGAM contained nine residues of cysteine. According to the tertiary structure, eight of these residues were capable of forming disulfide bridges, and one was a free cysteine (C319, as shown in Figure S4). Through the Ellman reaction, no free thiol groups were detected in the variants and AGAM when analyzed at 25 °C. However, after 5 min of incubation at 100 °C with GHCl, one thiol group was detected in AGAM, as well as in the variants G57A and D237G, but not in the variants C319T and C319A (Table 7).

In mass spectra of MALDI-TOF, only three of the seven cysteine residues were detected (Figure S4 and Tables S2–S7). MALDI-TOF analysis was performed on the protein bands obtained from SDS-PAGE using samples treated with 1% SDS and 5% 2-ME at 100 °C. Therefore, cysteine residues reduced from the disulfide bonds could be expected, either free or linked to acrylamide. AA sequence coverage for AGAM and the variants was 60–71% (Figure S4). Residues C261 and C269, which form a disulfide bridge C261–C269 in the spatial model of AGAM, were detected both free and linked to acrylamide (Figure S5a,b). Residue C604, but not residue C509 of the pair C509–C604, was also detected in both forms (Figure S5c,d). Cysteine residues from other disulfide bridges (C209–C212 and C221–C448) were not detected, probably because of the large molecular mass of the peptides, more than 4 kDa (Figure S6). Residue C319 was not detected for AGAM. Therefore, the presence of a free sulfohydryl group, as supposed from the analysis using the Ellman reaction, was not confirmed by MALDI-TOF. However, substitutions C319T and C319A were confirmed by fragmentation spectra (Figure S7a,b).

In SDS-PAGE, the variants C319T and C319A showed patterns similar to that of AGAM, so the formation of a protein band of 140 kDa cannot be attributed to disulfide bonding between two enzyme molecules through the sulfohydryl group of C319 (Figure S8). This was in contrast to *A. niger* ATCC 22343/65 GAM, for which dimer formation was reported due to the reformation of disulfide bonds in the absence of dithiothreitol or mercaptoethanol [67]. Also, thiol-disulfide interchange was reported for cysteine residues in *A. niger* (strain n.r.) GAM [50].

AGAM and the variants were similar in degree of glycosylation, being 15–16% as measured using the Dubois reaction (Table 7). Based on MALDI-TOF analysis, AA residues N170 and N394 were supposed to be glycosylated with high-mannose glycans of (Man)_5–8_(GlcNAc)_2_ (Table 7 and Tables S8–S13, Figures S9 and S10). The sites and structure of the *N*-linked glycans were putatively assigned by MALDI-TOF mass matching with Glycomod [33] for AGAM and the variants, in comparison with AGAM treated with Endo F1. However, fragmentation of glycopeptides was not performed in MALDI-TOF/TOF.

The high-mannose structure of *N*-linked glycans in AGAM was confirmed by the action of Endo F1 and PNGase F, but not by Endo F2 and F3 (Figure S11a). A decrease in the molecular weight (MW) of AGAM of approximately 5 kDa was observed after treatment with Endo F1 and PNGase F. Additionally, glycopeptides containing *N*-linked high-mannose glycans were not detected in the mass spectrum of AGAM treated with Endo F1 (Figures S9 and S10 for the sites N170 and N394, respectively). (HexNAc)_1_ was only detected linked to N394 (Figure S10a). A glycopeptide containing (HexNAc)_1_ linked to N170 was presented in the mass spectrum but with a low intensity (Figure S9a). Therefore, these two sites of *N*-glycosylation were assigned to be linked to high-mannose glycans in AGAM and the variants.

The data on AGAM glycosylation obtained were in accordance with a previous report of a small contribution of *N*-glycosylation to the MW of GAMs. The MWs of two *A. awamori* (strain n.r.) GAMs produced in *S. cerevisiae* C468 were reported to decrease after PNGase F treatment by 2.3 kDa [68] and 10 kDa [11]. The PNGase F treatment of *A. niveus* WFCC604 GAM, natively produced in *A. niveus* WFCC604, decreased MW by 9 kDa [66]. In the case of *A. niger* N402 GAM, the PNGase F treatment decreased MW by 2–5 kDa [64]. Additionally, overall glycosylation degrees of 13.7% and 16% were reported for *A. awamori* 2.B.361 U2/1 GAM [69] and *A. niger* (strain n.r.) GAMs [70], respectively.

As for the composition of *N*-linked glycans in fungal expression hosts, oligo- and high-mannose glycans have been reported, but no glycans with a hybrid or complex composition have been found [64]. Based on the crystal structure analysis of the CD of *A. awamori* var. X100 GAM, it was suggested that there are two *N*-glycosylation sites that contain high-mannose glycans [71]. The *N*-linked glycosylation pattern of *A. niger* N402 GAM has been reported to be (Man)_7–8_(GlcNAc)_2_ [64].

### 3.5. Assessment of AGAM Dimerization

In the case of AGAM, a decrease in MW of 5 kDa after the action of Endo F1 and PNGase F was close to the combined mass of 3.5 kDa for two glycans, (Man)_8_(GlcNAc)_2_, detected by MALDI-TOF. However, an MW of 140 kDa was obtained for AGAM and the variants in SDS-PAGE, while the theoretical weight of the protein chain of AGAM was 65.9 kDa, and the degree of glycosylation was 15–16%. Therefore, the difference in weights could not be explained by heavy glycosylation of the enzyme. Instead, it could be due to dimer formation or the effect of an O-glycosylated linker on glycoprotein migration in PAAG.

A dimer formation has been reported for *S. cerevisiae* sporulation GAM and *Rhizomucor pusillus* GAM. In the case of *S. cerevisiae* sporulation GAM, an unusual intracellular enzyme among generally extracellular GAMs, MWs of 190 kDa and 90 kDa were measured using sedimentation on a sucrose gradient for the crude extract and purified enzyme, respectively. *S. cerevisiae* sporulation GAM was supposed to form a dimer in the crude culture and dissociate into monomers during chromatographic purification [72]. *R. pusillus* GAM, which has a 36% AA sequence identity with *A. niger* AAT67041.1 GAM, formed a dimer with a MW of 114.5 kDa in solution, as shown by gel filtration, while a protein band of ~57 kDa was detected on SDS-PAGE, corresponding to a monomer [73].

**Figure 3:**
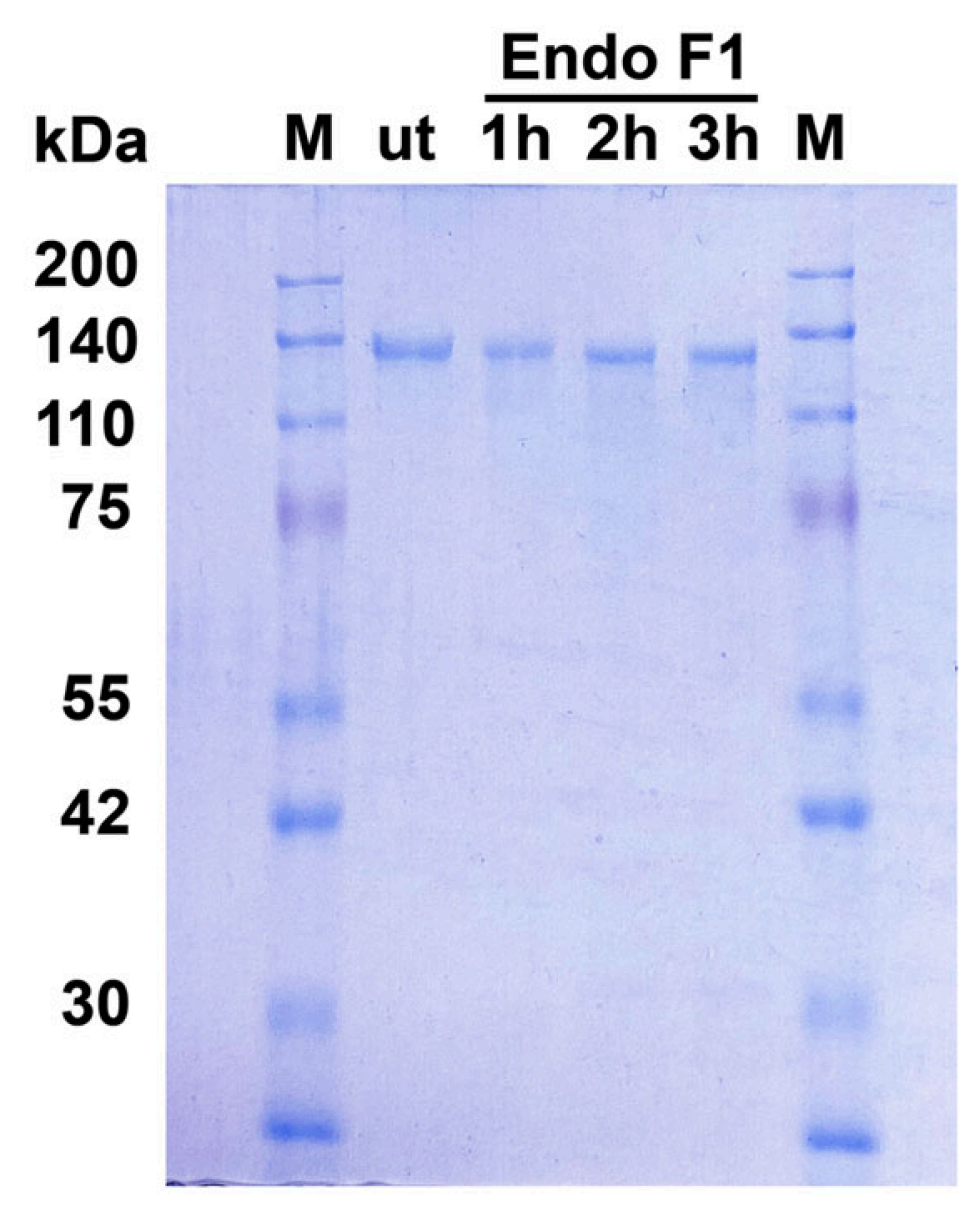
SDS-PAGE of AGAM treated with Endo F1 for 1, 2, and 3 h in comparison with untreated enzyme (ut). The side lanes correspond to a protein marker with molecular weights in kDa.

A pattern of deglycosylation could confirm the dimer structure of AGAM. The catalytic domain of AGAM contained two sites of *N*-linked glycosylation, and the dimer contained four such sites. When treated with Endo F1 or PNGase F for different periods of time, AGAM produces forms with different amounts of linked glycans. The number of bands on SDS-PAGE for partly deglycosylated AGAM corresponds to the number of glycosylation sites. However, this analysis was not successful. The theoretical difference in weight between glycoforms was ~1.5 kDa. Stairs of glycoforms were not distinguished on 4–8% polyacrylamide gels, even with thin protein bands, low sample dosage, and 1–24 h of treatment (Figure 3 and Figure S11). Difficulties in distinguishing glycoforms may be due to heterogeneity in *N*-glycosylation in AGAM. The glycans with different compositions, (HexNAc)_1_ and (Man)_5–8_(GlcNAc)_2_, were identified through MALDI-TOF analysis (Table 7), which revealed heterogeneity in MW. This heterogeneity in MW has previously been reported for *A. awamori* GAM (which is identical to *A. niger* GAM, strain n.r.) produced in *P. pastoris*, *S. cerevisiae*, and *A. niger* [47].

Characterization of glycoforms of full-length *A. awamori* (strain n.r.) GAM (consisting of CD, linker, and SBD) has been reported to be unsuccessful, similar to the present study on AGAM. However, two sites of *N*-linked glycosylation have been confirmed using only the CD [11].

Several enzymes with different domain structures were compared to AGAM using SDS-PAGE and Native-Blue electrophoresis. Specifically, *P. verruculosum* cellobiohydrolase I 66 kDa (PvCBHI-hm) is composed of a CD, a heavily glycosylated linker, and a cellulose-binding domain (Figure S12a). This enzyme also exists in a truncated form without SBD (PvCBHI-lm) (Figure S12b). *P. verruculosum* beta-glucosidase (PvBG) consists of two closely interacting structural domains (Figure S12d), while *A. niger* glucose oxidase (AnGOX) has one large domain (Figure S12c). All of these enzymes have multiple *N*-glycosylation sites within their catalytic domains.

In the Native-Blue electrophoresis, AGAM showed a protein band with MW 65 kDa, which was lower than the protein bands of PvCBHI-hm and PvBG, but similar to that of AnGOX (Figure S13a). The absence of bromophenol blue, a dye used for preparing samples for electrophoresis and affecting protein charge, as well as the presence of urea, a chemical compound that affects hydrophobic interactions, did not change the pattern of protein bands (Figure S13b,c). Similarly, in SDS-PAGE, the presence of urea also did not affect the protein band of AGAM (Figure S13d). However, the presence of SDS considerably changed the position of the protein bands of AGAM and PvCBHI-hm, while it did not affect the PvBG and AnGOX bands. Without heating at 100 °C, AGAM and PvCBHI-hm had similar MWs of 60 kDa. When heated at 100 °C, AGAM formed two protein bands of 70 and 140 kDa, whereas PvCBHI-hm formed a single 60 kDa band. If heated at 100 °C in the presence of 2-ME, AGAM formed a single band of 140 kDa, and PvCBHI-hm formed a band of 66 kDa (Figure S13e). This comparison of enzymes indicated that AGAM had a tightly packed spatial structure in solution at room temperature, with a major change in folding when heated to 100 °C.

**Figure 4:**
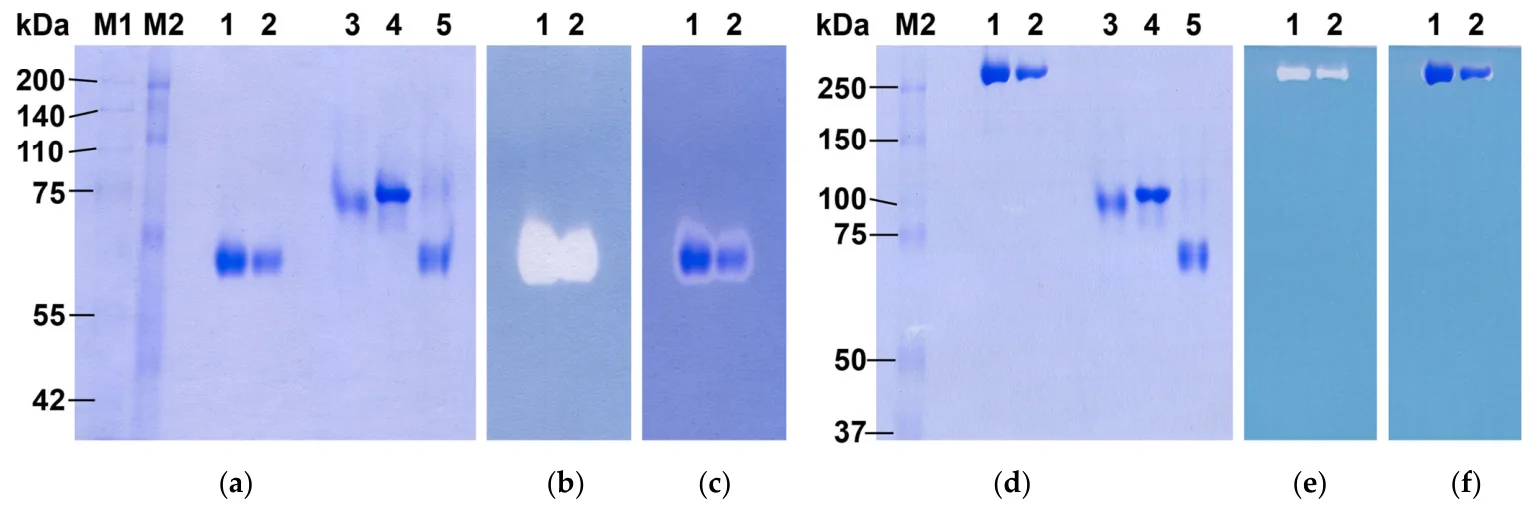
Native-Clean electrophoresis performed on PAAG (**a**–**c**) and PAAG containing 0.8% starch (**d**–**f**). Protein bands were stained with Coomassie blue in (**a**,**d**). After electrophoresis, the gel without starch was incubated with 0.8% starch solution and stained with I_2_ solution (**b**). The superposition of (**a**,**b**) is presented in (**c**). The gel containing starch was incubated in a 0.05 M NaAc buffer (pH 4.7) and stained with I_2_ solution (**e**). The superposition of (**d**,**e**) is presented in (**f**). The sample numbers are: 1—AGAM (6 μg), 2—AGAM (3 μg), 3—*P. verruculosum* cellobiohydrolase I-hm, 4—*P. verruculosum* beta-glucosidase, and 5—*A. niger* glucose oxidase. The lanes on the side correspond to protein markers with molecular weights in kDa.

The presence of soluble starch resulted in a multiple increase in the MW of AGAM. In Native-Clean electrophoresis, AGAM formed a single band of 65 kDa in the absence of starch (Figure 4a). When starch was added to the gel composition, the MW increased to more than 250 kDa (Figure 4d). It is worth noting that the position of the bands remained unchanged for other enzymes, such as PvCBHI-hm, PvBG, and AnGOX, which are not active towards starch. This confirmed that the addition of starch did not affect gel density or electrophoresis conditions.

The band of 65 kDa, obtained in the Native-Clean electrophoresis in the absence of starch, showed hydrolytic activity when placed in a solution of starch (Figure 4b). The other band, 250 kDa, obtained after electrophoresis using a mixture of PAAG and soluble starch, also hydrolyzed starch in the gel when placed in 0.05 M NaAc buffer (Figure 4e).

The increase in the molecular weight of AGAM observed in the presence of soluble starch could be explained by the formation of an enzyme dimer, enzyme-starch complex, or aggregate of several enzyme molecules with starch. The hypothesis of dimer formation is supported by the reported dimerization of *A. niger* (strain n.r.) GAM in the presence of a synthetic ligand, acarbose linked to β-cyclodextrin [1]. A clear band on PAAG with 0.8% starch was more likely to represent a quaternary structure with a fixed composition rather than an enzyme-starch complex or aggregate with a variable composition. Dimerization of AGAM may explain slower migration of bands in the presence of soluble starch and on SDS-PAGE. However, further investigation is needed to confirm this hypothesis.

### 3.6. Thermal Denaturation and Quaternary Structure

A low MW of AGAM in Native-Blue electrophoresis and SDS-PAGE (unheated) revealed that it forms a compact spatial structure in solution. This was in agreement with the reported study on the conformation of *A. niger* (strain n.r.) GAM; specifically, the glycosylated linker was proposed to form a highly structured and compact region between CD and SBD, leading to an oval conformation of the total enzyme in solution [1].

AGAM in a crude flask solution produced by *P. verruculosum* and as an individual enzyme was characterized by the same molecular weight in Native-Blue electrophoresis and SDS-PAGE (Figure S14). Therefore, AGAM did not change its quaternary structure after purification. This was in contrast to *S. cerevisiae* sporulation GAM, which was reported to form a dimer in crude culture and dissociate into monomers during chromatographic purification [72].

GAMs, which had considerably different MW under various conditions, showed a tendency towards a high T optimum of activity. At the same time, no association between the values of T optimum and time of half-inactivation at 60 °C was observed (Table 8). It should be noted that differences in host strains, degree of glycosylation, purification methods, and assays used for activity and thermostability measurements may confound the comparison. Nevertheless, dimerization of GAMs in the presence of starch or during thermodenaturation can be hypothesized; therefore, it can be used to select GAMs that demonstrate a high T optimum of activity towards starch.

The tertiary and quaternary structures of full-length *A. awamori* and *A. niger* GAMs have not been determined using crystallographic methods, so the relative positions of the CD and SBD, as well as the dimer formations of these GAMs, remain unknown. However, X-ray crystal structures of full-length *H. resinae* and *P. oxalicum* GAMs revealed multiple conformations of the SBD in relation to the CD [43]. Dimer formation has been proposed for *H. resinae* GAM based on PISA analysis [43] and for *A. niger* (strain n.r.) GAM based on small-angle X-ray scattering analysis [1].

Modeling of full-length AGAM using AlphaFold 2.0 allowed for confident structure prediction of the CD and SBD regions. The residues 3–441 in the CD and 517–616 in the SBD were predicted with pLDDT values ranging from 88.50 to 98.94 and from 73.69 to 98.56, respectively. However, the residues 474–506 in the linker region had much lower pLDDT values, ranging from 22.53 to 35.66 (Figure S15). Molecular dynamics simulations of AGAM showed a high degree of flexibility in the linker and SBD regions (Figure S16), which was consistent with multiple conformations of SBDs observed in crystal structures of *H. resinae* and *P. oxalicum* GAMs [43]. Only a single trajectory was carried out for AGAM, which limits the scope of the analysis.

In contrast to a monomer of AGAM, a dimer of the enzyme could not be modeled with AlphaFold 3.0 with confidence due to the absence of glycosylation in such a structure prediction. Therefore, a dimer of AGAM was modeled using an alignment with an annotated biological assembly of *H. resinae* GAM [43]. Molecular dynamics simulations of the AGAM dimer were performed at 30 and 100 °C. Simulations did not refute dimerization but showed that trajectories longer than 75 ns were needed for a reliable investigation (Figures S17–S19).

## 4. Discussion

AA substitution D237G, located on the surface of AGAM in the area of oligo- and polysaccharide binding, increased enzyme activity towards soluble starch by 1.3 times for an individual enzyme. This substitution also increased both *k_cat_* and *K_m_* by 1.3 and 1.2 times, respectively. This suggests that the residue D237 may be involved in substrate binding. The substitution D237G may cause weaker substrate binding, leading to a higher *K_m_*, as well as faster product dissociation from the active center, leading to an increase in *k_cat_*. For GAMs, the step of product dissociation from the active center [62,63] and the hydrolysis step, when the glycosidic bond in the substrate is broken [9], were proposed to determine the rate of the enzymatic reaction.

A homodimer formation of AGAM could be suggested in the presence of soluble starch. Similar dimerization has been reported for *A. niger* (strain n.r.) GAM in the presence of a synthetic ligand, acarbose linked to β-cyclodextrin [1]. At the same time, the observed changes in the migration of AGAM on PAAG may be due to the formation of an enzyme-starch complex or the aggregation of several enzyme molecules with starch. Further investigation is needed to determine the quaternary structure of AGAM and homologous enzymes.

T optimum temperature of the activity was reported in a wide range for different Aspergillus GAMs. T optimum was 70 °C for *A. niger* (strain n.r.) GAM [54], in contrast to optima of 50–65 °C for *A. niger* MTCC478 GAM [58], *A. niger* NIBGE-1 GAM [65], *A. niveus* WFCC604 GAM [66], and *A. flavus* GAM [2]. Differences in host strains, degree of glycosylation, purification methods, and assays for activity and thermostability measurements should be taken into account when comparing optima. However, a hypothesis that dimerization during starch hydrolysis provides higher T optima could be considered for the selection of GAMs operating under high temperatures.

Additionally, a 1.4-fold increase in activity was observed in a crude flask solution for the variant G57A when produced in *P. verruculosum* B1-537. A similar effect, 1.6-fold, was reported for *A. awamori* (strain n.r.) GAM with the substitution G57A when produced in *S. cerevisiae* C468 [5]. AA substitutions can affect not only the properties of individual enzymes, but also the activity in crude solutions obtained from fermentation, which affects the use of enzymes with AA substitutions in large-scale industrial production.

## Figures and Tables

**Table 1:** Amino acid substitutions and their effect on the spatial structure of AGAM.

AA Substitution	ConSurf −ln(f_sub_/f_in_)	ΔΔG				ΔΔS, ENCoM, kcal/mol/K	Predictions of Stabilization	Reference
		Maestro, kcal/mol	Rosetta, REU	FoldX, kcal/mol	SDM, kcal/mol			
V38I	−0.072	3.447	29.075	1.395	−2.29	−0.304	2	-
G57A	−2.060	−9.940	−0.296	−1.301	−2.15	−0.284	5	[5] and this study
Y115F	−1.701	2.240	0.683	−0.369	0.26	0.095	3	-
S118A	−0.405	8.205	−1.408	−0.163	−0.62	0.018	3	-
Y174F	−1.864	2.180	7.318	−0.023	0.46	0.054	3	[48,49]
F236I	−1.792	5.090	11.941	1.663	−0.52	0.475	1	-
D237G	−1.063	−4.431	−1.830	−0.607	−2.5	0.338	4	this study
S238G	−0.740	6.882	3.134	0.850	−0.16	0.048	1	-
S239G	−1.999	−1.358	0.460	0.056	−0.16	−0.111	3	-
F317Y	−1.634	1.753	5.070	1.890	−0.1	0.019	1	-
C319T	−0.426	−3.133	3.501	1.587	2.3	−0.179	4	this study
C319A	−0.031	−7.249	0.551	0.343	−0.33	0.248	2	[47,50] and this study
S398A	−0.370	2.510	−3.142	−0.115	−1.05	0.244	3	-
E408P	−3.063	−14.746	−1.980	−1.779	1.92	0.528	5	[3]

Stabilization is highlighted in green. The substitutions with the highest prediction score are underlined. For ConSurf, f_sub_ and f_in_ represent the frequencies of the substitution and the initial amino acid in the multiple sequence alignment. A negative value of −ln(f_sub_/f_in_) indicates a higher frequency of the substitution compared to the initial amino acid. For Maestro, Rosetta, and FoldX, a negative ΔΔG value indicates stabilization. For SDM, a positive ΔΔG value indicates stabilization. For ENCoM, a negative ΔΔS indicates lower molecular flexibility.

**Table 2:** Amino acid substitutions reported to improve properties of *A. awamori* (strain n.r.) GAM.

AA Substitution	Effect on Properties			Reference
	Enzyme Production ^a^	Activity ^b^	Thermostability	
G57A	1.6-fold increase	similar ^c^ activity	unchanged	[5]
C320A	5-fold decrease	modest decrease in activity, 1.4-fold decrease in *k_cat_*, and 1.4-fold increase in *K_m_*	1.5 °C increase in *T*_50_	[47,50]
E408P	not reported	similar ^c^ activity, 1.3-fold increase in *k_cat_*, and 1.2-fold increase in *K_m_*	6.7 °C decrease in *T_m_*	[3]

^a^ production host *S. cerevisiae* C468, ^b^ only the activity towards maltose was reported, ^c^ the difference was less than 1.05 times.

**Table 3:** Effect of amino acid substitutions on biochemical properties of AGAM.

AA Substitution	Activity, U/mg	*k_cat_*, s^−1^	*K_m_*, g∙L^−1^	*k_cat_*/*K_m_*, s^−1^∙g^−1^∙L	T Optimum, °C	pH Range of Near-Optimal Activity
-	35.8 ± 2.8	45.0 ± 0.8	0.68 ± 0.05	66.2 ± 6.0	70	3.0–4.0
G57A	39.6 ± 2.9	48.3 ± 0.5	0.66 ± 0.03	73.2 ± 4.1	70	3.0–4.0
D237G	46.3 ± 3.4	57.7 ± 0.4	0.79 ± 0.02	73.0 ± 2.4	70	3.0–4.0
C319T	38.8 ± 3.2	45.9 ± 0.3	0.63 ± 0.02	72.9 ± 2.8	70	3.0–4.0
C319A	35.7 ± 3.2	43.4 ± 0.6	0.61 ± 0.03	71.1 ± 4.5	70	3.0–4.0

**Table 4:** Effect of amino acid substitutions on thermostability of AGAM.

AA Substitution	*ΔT_m_* from Experimental Testing, °C	ConSurf, −ln(f_sub_/f_in_)	Predictions of ΔΔG				Prediction of ΔΔS, ENCoM, kcal/mol/K
			Maestro, kcal/mol	Rosetta, REU	FoldX, kcal/mol	SDM, kcal/mol	
G57A	−0.6 ± 0.2	−2.060	−9.940	−0.296	−1.301	−2.15	−0.284
C319T	−0.6 ± 0.2	−0.426	−3.133	3.501	1.587	2.3	−0.179
D237G	0.1 ± 0.2	−1.063	−4.431	−1.830	−0.607	−2.5	0.338
C319A	0.2 ± 0.2	−0.031	−7.249	0.551	0.343	−0.33	0.248

*T_m_* of AGAM was 70.7 ± 0.1 °C. Destabilization is highlighted in yellow; neutral effects are highlighted in green according to *T_m_* < 0.2 °C and −0.5 < ΔΔG < 0.5 kcal/mol.

**Table 5:** Amino acid residues in the active center of AGAM and those reported in *A. awamori* and *A. niger* GAMs.

AA Position in AGAM	Reported AA Position	Reported GAM	Effect on Activity	Reference
E178 and E399	E179 and E400	*A. awamori* ^a^	catalytic residues	[9,60]
E179	E180	*A. awamori* ^a^	induction of a conformational change in the bound substrate at the +1 subsite	[9,60]
Q181	N182	*A. awamori* ^a^	the nitrogen atoms of the Q and N side chains maintained enzyme activity	[11]
Y154	Y175	*A. niger* ^a^	location near the +3 subsite; Y175F decreased the activation energy in maltoheptaose hydrolysis by 0.705 kJ/mol and increased *k_cat_* and *K_m_* by 1.1–1.4 and 1.1–1.4 times, respectively ^b^	[48]
Y154	Y175	*A. awamori* ^a^	location near the +3 subsite; Y175F increased *k_cat_* and *K_m_* by 1.1–1.2 and 1.1–1.7 times, respectively ^b^	[49]

^a^ strain not reported, ^b^ hydrolysis of malto-oligosaccharides (degree of polymerization 4–7).

**Table 6:** Molecular weights of AGAM and Aspergillus GAMs determined using different methods.

Origin	Molecular Weight Under Native Conditions	Molecular Weight in SDS-PAGE	Reference
*A. awamori* VKPM F-1262 ^a^	65.9 kDa of the theoretical protein weight	140 kDa	this study
*A. niger* N402	70–80 kDa in native electroforesis	120–130 kDa	[64]
*A. niger* ^b^	57 kDa in MALDI-TOF, 62 kDa in native electroforesis	100–105 kDa	[54]
*A. niger* NIBGE-1	73 kDa in MALDI-TOF, 93 kDa in native electroforesis	93 kDa	[65]
*A. awamori* ^b^	80.37 kDa in MALDI-MS	94 kDa	[47]
*A. niveus* WFCC604	76 kDa in gel filtration	77 kDa	[66]

^a^ *A. awamori* VKPM F-1262 GAM with a substitution N181Q; ^b^ strain not reported.

**Table 7:** Structural characteristics of AGAM and the variants.

AA Substitution	Free Thiol Groups ^1^ Per Molecule 25 °C/100 °C (GHCl)	Degree of Glycosylation ^2^, %	Sites of *N*-Glycosylation ^3^	Structure of *N*-Linked Glycans ^3^		Theoretical Mass of *N*-Linked Glycans, kDa
-	0.01 ± 0.05/1.03 ± 0.05	16 ± 2	N170	(HexNAc)_1_,	(Man)_5–8_(GlcNAc)_2_	0.2–1.7
			N394	(HexNAc)_1_,	(Man)_5;8_(GlcNAc)_2_	0.2–1.7
G57A	0.01 ± 0.05/0.95 ± 0.04	16 ± 2	N170		(Man)_6–8_(GlcNAc)_2_	1.4–1.7
			N394	(HexNAc)_1_,	(Man)_8_(GlcNAc)_2_	0.2–1.7
D237G	0.01 ± 0.05/1.01 ± 0.04	16 ± 2	N170		(Man)_7–8_(GlcNAc)_2_	1.5–1.7
			N394	(HexNAc)_1_,	(Man)_8_(GlcNAc)_2_	0.2–1.7
C319T	0.01 ± 0.05/0.04 ± 0.05	15 ± 2	N170		(Man)_7_(GlcNAc)_2_	1.5
			N394	(HexNAc)_1_,	(Man)_8_(GlcNAc)_2_	0.2–1.7
C319A	0.01 ± 0.05/0.03 ± 0.05	15 ± 2	N170		(Man)_6–8_(GlcNAc)_2_	1.4–1.7
			N394	(HexNAc)_1_,	(Man)_8_(GlcNAc)_2_	0.2–1.7

^1^ Ellman reaction, ^2^ Dubois reaction for total (both *N*- and *O*-) glycosylation, ^3^ MALDI-TOF mass matching with Glycomod for *N*-glycosylation.

**Table 8:** Biochemical properties of AGAM and Aspergillus GAMs.

Origin	T Optimum, °C	Time of Half-Inactivation at 60 °C, min	Molecular Weight Under Non-Denaturing Conditions, kDa	Molecular Weight in SDS-PAGE, kDa	Reference
*A. awamori* VKPM F-1262 ^a^	70	210	65	140	this study
*A. niger* N402	n.r.	n.r.	70–80	120–130	[64]
*A. niger* ^b^	70	25	62	100–105	[54]
*A. niger* MTCC478	60	70	n.r.	75	[58]
*A. niger* NIBGE-1	60	n.r.	93	93	[65]
*A. niveus* WFCC604	65	total activity after 240 min	n.r.	77	[66]
*A. flavus* ^b^	50	>100	n.r.	63	[2]

^a^ *A. awamori* VKPM F-1262 GAM with a substitution N181Q, ^b^ strain not reported, n.r.—not reported.

## Data Availability

The original contributions presented in this study are included in the article/Supplementary Materials. Further inquiries can be directed to the corresponding author.

## Supplementary Materials

The following supporting information can be downloaded at https://www.mdpi.com/article/10.3390/biotech15040074/s1, Figure S1: Enzyme activity towards soluble starch in cultures after 6 days of cultivation in plates sorted from the highest value to the lowest. Figure S2: Enzyme activity towards soluble starch in cultures after 6 days of cultivation in flasks. Figure S3: SDS-PAGE of crude cultures after 6 days of cultivation in flasks (a) and purified enzymes (b–d) for AGAM and the variants G57A, D237G, C319T, and C319A (1, 2, 3, 4, and 5, respectively). Figure S4: Peptides were detected using MALDI-TOF in the AA sequences of AGAM, dAGAM (after deglycosylation with Endo F1), and the variants. Figure S5: Peaks were detected using MALDI-TOF for peptides containing cysteine residues in the AA sequences of AGAM, dAGAM (after treatment with Endo F1), and the variants. Figure S6: Theoretical peptides resulting from the cleavage of AGAM by trypsin in the mass range of *m*/*z* 500–6500. Figure S7: Spectra of fragmentation obtained through MALDI-TOF/TOF for the variants C319T (a) and C319A (b). Figure S8: SDS-PAGE of AGAM (a) and the variants C319T and C319A (b). Figure S9: Glycosylation linked to the site N170 in AGAM, dAGAM (after treatment with Endo F1), and the variants. Figure S10: Glycosylation linked to the site N394 in AGAM, dAGAM (after treatment with Endo F1), and the variants. Figure S11: SDS-PAGE of AGAM treated with Endo F1, Endo F2, and Endo F3, as well as PNGase F. Figure S12: Spatial structures of *P. verruculosum* cellobiohydrolase I in full-length (a) and truncated (b) forms (PvCBHI-hm and PvCBHI-lm, respectively), *A. niger* glucose oxidase (AnGOX) (c), *P. verruculosum* beta-glucosidase (PvBG) (d), and AGAM (e). Figure S13: Native-Blue (a), Native-Clean (b), Native-Blue (with 0.015 M urea in PAAG) (c), and SDS-PAGE (d and e) of AGAM, *P. verruculosum* cellobiohydrolase I in full-length and truncated forms (PvCBHI-hm and PvCBHI-lm, respectively), *P. verruculosum* beta-glucosidase (PvBG), and *A. niger* glucose oxidase (AnGOX). Figure S14: SDS-PAGE (a) and Native-Blue (b) of a crude flask solution (marked as “C”) and a purified enzyme (marked as “E”) of AGAM. Figure S15: A full-length model AGAM constructed using AlphaFold 2.0, with an accuracy of pLDDT = 92.6 and pTM = 0.772. Figure S16: MD simulations of AGAM as a monomer and glycoprotein at 30 °C for 75 ns. The model of AGAM was created using AlphaFold 2.0, and glycans were added using Glycan Reader and Modeler. Figure S17: MD simulations of AGAM as a dimer and glycoprotein at 30 °C for 10 ns. Figure S18: MD simulations of AGAM as a dimer and glycoprotein at 100 °C for 75 ns. Figure S19: A dimer of AGAM obtained through MD simulations at 30 °C (a) and 100 °C (b). Table S1: Oligonucleotide primers used for side-directed mutagenesis. Table S2: Peptides detected using MALDI-TOF for AGAM. Table S3: Peptides detected using MALDI-TOF for deglycosylated form of AGAM. Table S4: Peptides detected using MALDI-TOF for the variant G81A (G57A in the mature enzyme without a signal peptide). Table S5: Peptides detected using MALDI-TOF for the variant D261G (D237G in the mature enzyme without a signal peptide). Table S6: Peptides detected using MALDI-TOF for the variant C343T (C319T in the mature enzyme without a signal peptide). Table S7: Peptides detected using MALDI-TOF for the variant C343A (C319A in the mature enzyme without a signal peptide). Table S8: Glycopeptides detected using MALDI-TOF for AGAM. Table S9: Glycopeptides detected using MALDI-TOF for deglycosylated form of AGAM. Table S10: Glycopeptides detected using MALDI-TOF for the variant G81A (G57A in the mature enzyme without a signal peptide). Table S11: Glycopeptides detected using MALDI-TOF for the variant D261G (D237G in the mature enzyme without a signal peptide). Table S12: Glycopeptides detected using MALDI-TOF for the variant C343T (C319T in the mature enzyme without a signal peptide). Table S13: Glycopeptides detected using MALDI-TOF for the variant C343A (C319A in the mature enzyme without a signal peptide).

## Abbreviations

The following abbreviations are used in this manuscript:

AA	Amino acid
AGAM	*A. awamori* VKPM F-1262 glucoamylase with the substitution N181Q
BPB	Bromophenol blue
CD	Catalytic domain
GAM	Glucoamylase
GHCl	Guanidine hydrochloride
Glc	Glucose
GlcNAc	N-acetylglucosamine
MALDI-TOF/TOF	Matrix-Assisted Laser Desorption/Ionization Time-of-Flight/Time-of-Flight
Man	Mannose
MD	Molecular dynamics
2-ME	2-Mercaptoethanol
MW	Molecular weight
n.r.	Not reported
PAAG	Polyacrylamide gel
SBD	Starch-binding domain
SDS	Sodium dodecyl sulfate
