# Supplementary material for: Rational Design of Aspergillus awamori Glucoamylase for Activity Improvement
Source: BioTech (Basel). 2026 Sep 2;15(4):74. doi: 10.3390/biotech15040074 (PMC13600003; doi:10.3390/biotech15040074)
Supplement: Supplementary file 1 [file biotech-15-00074-s001.zip › biotech-4467952-supplementary.pdf]

Supplementary material

Rational design of *Aspergillus awamori* glucoamylase for activity improvement

Table S1. Oligonucleotide primers used for side-directed mutagenesis.

Amino acid (AA) substitution	Fwd-primer	Rev-primer
G57A	5'-cgactctgctctcgtcatc-3'	5'-gatgacgagagcagagtcg-3'
D237G	5'-ccaacttcggtagcagc-3'	5'-gctgctaccgaagttgg-3'
C319T	5'-ggttcctgactaccttgg-3'	5'-ccaaggtagtcaggaacc-3'
C319A	5'-gttcctggccaccttg-3'	5'-caaggtggccaggaac-3'

Table S2. Peptides detected using MALDI-TOF for AGAM. Cys_PAM is a cysteine residue modified with acrylamide to form propionamide cysteine; TPO and MSO are tryptophan and methionine in the oxidized form, respectively. The numbering is for the amino acid sequence including a signal peptide.

user mass	DB mass	Δmass (daltons)	peptide	position	modifications	missed cleavages
1633.773	1633.818	0.044	(R)/ATLDSWLSNEATVAR/(T)	25-39		0
4102.072	4101.946	-0.125	(R)/TAILNNIGADGAWVSGADSG IVVASPSTDNPDYFYTWTR/(D)	40-78		0
849.487	849.483	-0.004	(K)/SLVDLFR/(N)	86-92		0
3929.079	3928.941	-0.138	(R)/NGDTSLLSTIEHYISSQAII QGVSNPSGDLSSGGLGEPK/(F)	93-131		0
1983.902	1983.931	0.028	(K)/FNVDETAYTGSWGRPQR/(D)	132-148		0
1999.919	1999.926	0.006	(K)/FNVDETAYTGS<u>W</u>GRPQR/(D)	132-148	TPO	0
3238.724	3238.624	-0.099	(R)/ATAMIDFGNWLIDNGYTSVATDIVWPLVR/(N)	155-183		0
3254.723	3254.619	-0.104	(R)/ATAMIDFGN<u>W</u>LIDNGYTSVATDIVWPLVR/(N)	155-183	TPO	0
3254.723	3254.619	-0.104	(R)/ATA<u>M</u>IDFGNWLIDNGYTSVATDIVWPLVR/(N)	155-183	MSO	0
3254.723	3254.619	-0.104	(R)/ATAMIDFGNWLIDNGYTSVATDIV<u>W</u>PLVR/(N)	155-183	TPO	0
3508.739	3508.574	-0.164	(R)/SGKDANTLLGSIHTFDPEAG<u>C</u>DDSTFQP<u>C</u>SPR/ (A)	265-296	CYS_PAM CYS_PAM	1
1485.750	1485.781	0.030	(R)/ALANHKEVVDSFR/(S)	297-309		1
1966.939	1966.972	0.032	(R)/SIYTINDGLSDSEAVAVGR/(Y)	310-328		0
4325.170	4325.051	-0.119	(K)/QGELEITDVSLDFFQALYSSAATGTYSSSSSTY SSIVDAVK/(T)	361-401		0
1708.824	1708.865	0.041	(R)/DLTWSYAALLTANNR/(R)	437-451		0
1864.927	1864.966	0.039	(R)/DLTWSYAALLTANNRR/(N)	437-452		1
2652.326	2652.287	-0.039	(K)/YTSSNPLWYVTVTLPAGESF EYK/(F)	580-602		0
2194.000	2194.005	0.004	(K)/FIRVESDDTVEWESDPNR/(E)	603-620		1
4519.028	4519.063	0.034	(K)/FIRVESDDTVEWESDPNREYTVPQT<u>C</u>GESTVT VTDTWR	603-640	CYS_PAM	2
1777.701	1777.751	0.050	(R)/VESDDTVEWESDPNR/(E)	606-620		0
2344.074	2344.076	0.002	(R)/EYTVPQT<u>C</u>GESTVTVTDTWR	621-640	CYS_PAM	0

Table S3. Peptides detected using MALDI-TOF for deglycosylated form of AGAM. Cys_PAM is a cysteine residue modified with acrylamide to form propionamide cysteine; TPO and MSO are tryptophan and methionine in the oxidized form, respectively. The numbering is for the amino acid sequence including a signal peptide.

user mass	DB mass	Δmass (daltons)	peptide	position	modifications	missed cleavages
1633.822	1633.818	-0.004	(R)/ATLDSWLSNEATVAR/(T)	25-39		0
4101.905	4101.946	0.041	(R)/TAILNNIGADGAWVSGADSGIVVASPSTDNPD YFYTWTR/(D)	40-78		0
849.564	849.483	-0.081	(K)/SLVDLFR/(N)	86-92		0
3928.967	3928.941	-0.026	(R)/NGDTSLLSTIEHYISSQAIIQGVSNPSGDLSSGG LGEPK/(F)	93-131		0
1983.942	1983.931	-0.011	(K)/FNVDETAYTGSWGRPQR/(D)	132-148		0
1999.963	1999.926	-0.037	(K)/FNVDETAYTGSWGRPQR/(D)	132-148	TPO	0
2593.297	2593.254	-0.042	(K)/FNVDETAYTGSWGRPQRDGP ALR/(A)	132-154		1
3238.685	3238.624	-0.060	(R)/ATAMIDFGNWLIDNGYTSVATDIVWPLVR/(N)	155-183		0
3254.725	3254.619	-0.106	(R)/ATAMIDFGNWLIDNGYTSVATDIVWPLVR/(N)	155-183	TPO	0
3254.725	3254.619	-0.106	(R)/ATAMIDFGNWLIDNGYTSVATDIVWPLVR/(N)	155-183	TPO	0
3254.725	3254.619	-0.106	(R)/ATAMIDFGNWLIDNGYTSVATDIVWPLVR/(N)	155-183	MSO	0
3508.676	3508.574	-0.101	(R)/SGKDANTLLGSIHTFDPEAGCDDSTFQPCSPR/ (A)	265-296	CYS_PAM CYS_PAM	1
1485.793	1485.781	-0.012	(R)/ALANHKEVVDSFR/(S)	297-309		1
1966.976	1966.972	-0.004	(R)/SIYTINDGLSDSEAVAVGR/(Y)	310-328		0
1708.868	1708.865	-0.002	(R)/DLTWSYAALLTANNR/(R)	437-451		0
1864.965	1864.966	0.001	(R)/DLTWSYAALLTANNRR/(N)	437-452		1
2194.046	2194.005	-0.041	(K)/FIRVESDDTVEWESDPNR/(E)	603-620		1
4518.819	4519.063	0.243	(K)/FIRVESDDTVEWESDPNREYTVPQTCGESTVT VTDTWR	603-640	CYS_PAM	2
2344.120	2344.076	-0.043	(R)/EYTVPQTCGESTVTVTDTWR	621-640	CYS_PAM	0

Table S4. Peptides detected using MALDI-TOF for the variant G81A (G57A in the mature enzyme without a signal peptide). Cys_PAM is a cysteine residue modified with acrylamide to form propionamide cysteine; TPO and MSO are tryptophan and methionine in the oxidized form, respectively. The numbering is for the amino acid sequence including a signal peptide.

user mass	DB mass	Δmass (daltons)	peptide	position	modifications	missed cleavages
1633.850	1633.818	-0.032	(R)/ATLDSWLSNEATVAR/(T)	25-39		0
1649.838	1649.813	-0.025	(R)/ATLDSWLSNEATVAR/(T)	25-39	TPO	0
4101.902	4101.946	0.044	(R)/TAILNNIGADGAWVSGADSG IVVASPSTDNPDYFYTWTR/(D)	40-78		0
4117.861	4117.941	0.080	(R)/TAILNNIGADGAWVSGADSG IVVASPSTDNPDYFYTWTR/(D)	40-78	TPO	0
4117.861	4117.941	0.080	(R)/TAILNNIGADGAWVSGADSG IVVASPSTDNPDYFYTWTR/(D)	40-78	TPO	0
745.411	745.445	0.034	(R)/DSALVIK/(S)	79-85		0
849.465	849.483	0.017	(K)/SLVDLFR/(N)	86-92		0
3928.935	3928.941	0.005	(R)/NGDTSLLSTIEHYISSQAIIQGVSNPSGDLSSGG LGEPK/(F)	93-131		0
1983.977	1983.931	-0.046	(K)/FNVDETAYTGSWGRPQR/(D)	132-148		0
1999.989	1999.926	-0.063	(K)/FNVDETAYTGSWGRPQR/(D)	132-148	TPO	0
628.331	628.341	0.010	(R)/DGPALR/(A)	149-154		0
3238.640	3238.624	-0.015	(R)/ATAMIDFGNWLIDNGYTSVATDIVWPLVR/(N)	155-183		0
3254.642	3254.619	-0.023	(R)/ATAMIDFGNWLIDNGYTSVATDIVWPLVR/(N)	155-183	TPO	0
3254.642	3254.619	-0.023	(R)/ATAMIDFGNWLIDNGYTSVATDIVWPLVR/(N)	155-183	MSO	0
3254.642	3254.619	-0.023	(R)/ATAMIDFGNWLIDNGYTSVATDIVWPLVR/(N)	155-183	TPO	0
3508.583	3508.574	-0.008	(R)/SGKDANTLLGSIHTFDPEAGCDDSTFQPCSPR/ (A)	265-296	CYS_PAM CYS_PAM	1
3165.423	3165.389	-0.034	(K)/DANTLLGSIHTFDPEAGCDDSTFQPCSPR/(A)	268-296	CYS_PAM	0
3165.423	3165.389	-0.034	(K)/DANTLLGSIHTFDPEAGCDDSTFQPCSPR/(A)	268-296	CYS_PAM	0
653.365	653.373	0.007	(R)/ALANHK/(E)	297-302		0
1485.804	1485.781	-0.023	(R)/ALANHKEVVDSFR/(S)	297-309		1
851.420	851.426	0.005	(K)/EVVDSFR/(S)	303-309		0
1967.016	1966.972	-0.044	(R)/SIYTINDGLSDSEAVAVGR/(Y)	310-328		0

935.434	935.443	0.008	(K)/SSGEELSAR/(D)	428-436		0
1708.898	1708.865	-0.032	(R)/DLTWSYAALLTANNR/(R)	437-451		0
1865.001	1864.966	-0.034	(R)/DLTWSYAALLTANNRR/(N)	437-452		1
1880.972	1880.961	-0.010	(R)/DLTWSYAALLTANNRR/(N)	437-452	TPO	1
2652.323	2652.287	-0.036	(K)/YTSSNPLWYVTVTLPAGESFEYK/(F)	580-602		0
2194.048	2194.005	-0.043	(K)/FIRVESDDTVEWESDPNR/(E)	603-620		1
4518.994	4519.063	0.068	(K)/FIRVESDDTVEWESDPNREYTVPQTCGESTVT VTDTWR	603-640	CYS_PAM	2
1777.787	1777.751	-0.035	(R)/VESDDTVEWESDPNR/(E)	606-620		0
2273.103	2273.039	-0.063	(R)/EYTVPQTCGESTVTVTDTWR	621-640		0
2344.136	2344.076	-0.059	(R)/EYTVPQTCGESTVTVTDTWR	621-640	CYS_PAM	0

Table S5. Peptides detected using MALDI-TOF for the variant D261G (D237G in the mature enzyme without a signal peptide). Cys_PAM is a cysteine residue modified with acrylamide to form propionamide cysteine; TPO and MSO are tryptophan and methionine in the oxidized form, respectively. The numbering is for the amino acid sequence including a signal peptide.

user mass	DB mass	Δmass (daltons)	peptide	position	modifications	missed cleavages
1633.842	1633.818	-0.024	(R)/ATLDSWLSNEATVAR/(T)	25-39		0
1649.831	1649.813	-0.018	(R)/ATLDSWLSNEATVAR/(T)	25-39	TPO	0
4101.915	4101.946	0.031	(R)/TAILNNIGADGAWVSGADSGIVVASPSTDNP DYFYTWTR/(D)	40-78		0
4117.860	4117.941	0.081	(R)/TAILNNIGADGAWVSGADSGIVVASPSTDNP DYFYTWTR/(D)	40-78	TPO	0
4117.860	4117.941	0.081	(R)/TAILNNIGADGAWVSGADSGIVVASPSTDNP DYFYTWTR/(D)	40-78	TPO	0
731.398	731.430	0.031	(R)/DSGLVIK/(S)	79-85		0
849.470	849.483	0.012	(K)/SLVDLFR/(N)	86-92		0
3928.933	3928.941	0.007	(R)/NGDTSLLSTIEHYISSQAIIQGVSNPSGDLSSG GLGEPK/(F)	93-131		0
1983.962	1983.931	-0.031	(K)/FNVDETAYTGSWGRPQR/(D)	132-148		0
628.331	628.341	0.010	(R)/DGPALR/(A)	149-154		0
3254.628	3254.619	-0.009	(R)/ATAMIDFGNWLIDNGYTSVATDIVWPLVR/ (N)	155-183	MSO	0
3254.628	3254.619	-0.009	(R)/ATAMIDFGNWLIDNGYTSVATDIVWPLVR/ (N)	155-183	TPO	0
3254.628	3254.619	-0.009	(R)/ATAMIDFGNWLIDNGYTSVATDIVWPLVR/ (N)	155-183	TPO	0
4051.852	4051.889	0.036	(R)/NDLSYVAQYWNQTGYDLWEEVQGSSFFTIA VQHR/(A)	184-217		0
3508.587	3508.574	-0.012	(R)/SGKDANTLLGSIHTFDPEAGCDDSTFQPCSPR /(A)	265-296	CYS_PAM CYS_PAM	1
3165.418	3165.389	-0.029	(K)/DANTLLGSIHTFDPEAGCDDSTFQPCSPR/(A)	268-296	CYS_PAM	0
3165.418	3165.389	-0.029	(K)/DANTLLGSIHTFDPEAGCDDSTFQPCSPR/(A)	268-296	CYS_PAM	0
3236.463	3236.426	-0.037	(K)/DANTLLGSIHTFDPEAGCDDSTFQPCSPR/(A)	268-296	CYS_PAM CYS_PAM	0
1485.800	1485.781	-0.019	(R)/ALANHKEVVDSFR/(S)	297-309		1
1966.996	1966.972	-0.024	(R)/SIYTINDGLSDSEAVAVGR/(Y)	310-328		0
4325.025	4325.051	0.025	(K)/QGELEITDVSLDFFQALYSSAATGTYSSSSST YSSIVDAV K/(T)	361-401		0
2757.305	2757.300	-0.004	(K)/TFADGFVSIVETHAASNGSLSEQFDK/(S)	402-427		0
3673.678	3673.725	0.047	(K)/TFADGFVSIVETHAASNGSLSEQFDKSSGEEL SAR/(D)	402-436		1
1708.888	1708.865	-0.022	(R)/DLTWSYAALLTANNR/(R)	437-451		0
1864.987	1864.966	-0.020	(R)/DLTWSYAALLTANNRR/(N)	437-452		1
2652.316	2652.287	-0.029	(K)/YTSSNPLWYVTVTLPAGESF EYK/(F)	580-602		0
2194.046	2194.005	-0.041	(K)/FIRVESDDTVEWESDPNR/(E)	603-620		1
4519.007	4519.063	0.055	(K)/FIRVESDDTVEWESDPNREYTVPQTCGESTV TVTDTWR	603-640	CYS_PAM	2
1777.776	1777.751	-0.024	(R)/VESDDTVEWESDPNR/(E)	606-620		0
4031.766	4031.772	0.006	(R)/VESDDTVEWESDPNREYTVPQTCGESTVTVT DTWR	606-640		1
2273.085	2273.039	-0.045	(R)/EYTVPQTCGESTVTVTDTWR	621-640		0
2344.135	2344.076	-0.058	(R)/EYTVPQTCGESTVTVTDTWR	621-640	CYS_PAM	0

Table S6. Peptides detected using MALDI-TOF for the variant C343T (C319T in the mature enzyme without a signal peptide). Cys_PAM is a cysteine residue modified with acrylamide to form propionamide cysteine; TPO and MSO are tryptophan and methionine in the oxidized form, respectively. The numbering is for the amino acid sequence including a signal peptide.

user mass	DB mass	Δmass (daltons)	peptide	position	modifications	missed cleavages
1633.817	1633.818	0.001	(R)/ATLDSWLSNEATVAR/(T)	25-39		0
1649.792	1649.813	0.020	(R)/ATLDSWLSNEATVAR/(T)	25-39	TPO	0
4101.930	4101.946	0.016	(R)/TAILNNIGADGAWVSGADSGIVVASPSTDNP DYFYTWTR/(D)	40-78		0
849.476	849.483	0.006	(K)/SLVDLFR/(N)	86-92		0
3928.951	3928.941	-0.010	(R)/NGDTSLLSTIEHYISSQAIIQGVSNPSGDLSSG GLGEPK/(F)	93-131		0
1983.949	1983.931	-0.018	(K)/FNVDETAYTGSWGRPQR/(D)	132-148		0
1999.964	1999.926	-0.038	(K)/FNVDETAYTGSWGRPQR/(D)	132-148	TPO	0
628.335	628.341	0.006	(R)/DGPALR/(A)	149-154		0
3238.607	3238.624	0.017	(R)/ATAMIDFGNWLIDNGYTSVATDIVWPLVR/ (N)	155-183		0
3254.628	3254.619	-0.009	(R)/ATAMIDFGNWLIDNGYTSVATDIVWPLVR/ (N)	155-183	TPO	0
3254.628	3254.619	-0.009	(R)/ATAMIDFGNWLIDNGYTSVATDIVWPLVR/ (N)	155-183	TPO	0
3254.628	3254.619	-0.009	(R)/ATAMIDFGNWLIDNGYTSVATDIVWPLVR/ (N)	155-183	MSO	0
3508.591	3508.574	-0.016	(R)/SGKDANTLLGSIHTFDPEAGCDDSTFQPCSPR /(A)	265-296	CYS_PAM CYS_PAM	1
1485.778	1485.781	0.002	(R)/ALANHKEVVDSFR/(S)	297-309		1
851.429	851.426	-0.003	(K)/EVVDSFR/(S)	303-309		0
1966.989	1966.972	-0.017	(R)/SIYTINDGLSDSEAVAVGR/(Y)	310-328		0
935.437	935.443	0.005	(K)/SSGEELSAR/(D)	428-436		0
1708.868	1708.865	-0.002	(R)/DLTWSYAALLTANNR/(R)	437-451		0
1864.972	1864.966	-0.005	(R)/DLTWSYAALLTANNRR/(N)	437-452		1
2652.298	2652.287	-0.011	(K)/YTSSNPLWYVTVTLPAGESFEYK/(F)	580-602		0
2194.023	2194.005	-0.018	(K)/FIRVESDDTVEWESDPNR/(E)	603-620		1
4519.007	4519.063	0.055	(K)/FIRVESDDTVEWESDPNREYTVPQTCGESTV TVTDTWR	603-640	CYS_PAM	2
1777.759	1777.751	-0.007	(R)/VESDDTVEWESDPNR/(E)	606-620		0
2344.112	2344.076	-0.035	(R)/EYTVPQTCGESTVTVTDTWR	621-640	CYS_PAM	0

Table S7. Peptides detected using MALDI-TOF for the variant C343A (C319A in the mature enzyme without a signal peptide). Cys_PAM is a cysteine residue modified with acrylamide to form propionamide cysteine; TPO and MSO are tryptophan and methionine in the oxidized form, respectively. The numbering is for the amino acid sequence including a signal peptide.

user mass	DB mass	Δmass (daltons)	peptide	position	modifications	missed cleavages
1633.841	1633.818	-0.023	(R)/ATLDSWLSNEATVAR/(T)	25-39		0
1649.833	1649.813	-0.020	(R)/ATLDSWLSNEATVAR/(T)	25-39	TPO	0
4101.911	4101.946	0.035	(R)/TAILNNIGADGAWVSGADSGIVVASPSTDNPD YFYTWTR/(D)	40-78		0
849.469	849.483	0.013	(K)/SLVDLFR/(N)	86-92		0
3928.939	3928.941	0.001	(R)/NGDTSLLSTIEHYISSQAIIQGVSNPSGDLSSGG LGEPK/(F)	93-131		0
1983.967	1983.931	-0.036	(K)/FNVDETAYTGSWGRPQR/(D)	132-148		0
1999.971	1999.926	-0.045	(K)/FNVDETAYTGSWGRPQR/(D)	132-148	TPO	0
3847.681	3847.948	0.266	(R)/DGPALRATAMIDFGNWLIDNGYTSVATDIVW PLVR/(N)	149-183		1
3238.635	3238.624	-0.010	(R)/ATAMIDFGNWLIDNGYTSVATDIVWPLVR/(N)	155-183		0
3254.637	3254.619	-0.018	(R)/ATAMIDFGNWLIDNGYTSVATDIVWPLVR/(N)	155-183	TPO	0
3254.637	3254.619	-0.018	(R)/ATAMIDFGNWLIDNGYTSVATDIVWPLVR/(N)	155-183	MSO	0
3254.637	3254.619	-0.018	(R)/ATAMIDFGNWLIDNGYTSVATDIVWPLVR/(N)	155-183	TPO	0
3508.575	3508.574	0.000	(R)/SGKDANTLLGSIHTFDPEAGCDDSTFQPCSPR/ (A)	265-296	CYS_PAM CYS_PAM	1
3165.415	3165.389	-0.026	(K)/DANTLLGSIHTFDPEAGCDDSTFQPCSPR/(A)	268-296	CYS_PAM	0
3165.415	3165.389	-0.026	(K)/DANTLLGSIHTFDPEAGCDDSTFQPCSPR/(A)	268-296	CYS_PAM	0
1485.811	1485.781	-0.030	(R)/ALANHKEVVDSFR/(S)	297-309		1
851.412	851.426	0.013	(K)/EVVDSFR/(S)	303-309		0
1967.009	1966.972	-0.037	(R)/SIYTINDGLSDSEAVAVGR/(Y)	310-328		0
3815.745	3815.754	0.008	(R)/YPEDSYYNGNPWFLATLAAAEQLYDALYQW DK/(Q)	329-360		0
3847.681	3847.744	0.062	(R)/YPEDSYYNGNPWFLATLAAAEQLYDALYQW DK/(Q)	329-360	TPO TPO	0
4324.987	4325.051	0.063	(K)/QGELEITDVSLDFFQALYSSAATGTYSSSSSTY SSIVDAVK/(T)	361-401		0
935.429	935.443	0.013	(K)/SSGEELSAR/(D)	428-436		0
1708.893	1708.865	-0.027	(R)/DLTWSYAALLTANNR/(R)	437-451		0
1864.994	1864.966	-0.027	(R)/DLTWSYAALLTANNRR/(N)	437-452		1
2652.306	2652.287	-0.019	(K)/YTSSNPLWYVTVTLPAGESFEYK/(F)	580-602		0
2194.042	2194.005	-0.037	(K)/FIRVESDDTVEWESDPNR/(E)	603-620		1
4518.996	4519.063	0.066	(K)/FIRVESDDTVEWESDPNREYTVPQTCGESTVT VTDTWR	603-640	CYS_PAM	2
1777.785	1777.751	-0.033	(R)/VESDDTVEWESDPNR/(E)	606-620		0
2344.132	2344.076	-0.055	(R)/EYTVPQTCGESTVTVTDTWR	621-640	CYS_PAM	0

Table S8. Glycopeptides detected using MALDI-TOF for AGAM. The numbering is for the amino acid sequence including a signal peptide.

user mass	theor. glycopeptide mass	Δmass (daltons)	glycan structure	peptide mass [M]	peptide sequence
2960.462	2960.379	0.083	$(HexNAc)_1$	2756.293	402-427 TFADGFVSIVETHAASNGSLSEQFDK
3973.830	3973.723	0.107	$(Hex)_2$ + $(Man)_3(GlcNAc)_2$	2756.293	402-427 TFADGFVSIVETHAASNGSLSEQFDK
4459.980	4459.881	0.099	$(Hex)_5$ + $(Man)_3(GlcNAc)_2$	2756.293	402-427 TFADGFVSIVETHAASNGSLSEQFDK
3876.914	3876.804	0.110	$(HexNAc)_1$	3672.718	402-436 TFADGFVSIVETHAASNGSLSEQFDKSSGE ELSAR (1 missed cleavage)
4890.165	4890.148	0.017	$(Hex)_2$ + $(Man)_3(GlcNAc)_2$	3672.718	402-436 TFADGFVSIVETHAASNGSLSEQFDKSSGE ELSAR (1 missed cleavage)
5376.222	5376.306	-0.083	$(Hex)_5$ + $(Man)_3(GlcNAc)_2$	3672.718	402-436 TFADGFVSIVETHAASNGSLSEQFDKSSGE ELSAR (1 missed cleavage)
4255.029	4254.968	0.061	$(HexNAc)_1$	4050.881	184-217 NDLSYVAQYWNQTGYDLWEEVQGSSFFTIAVQHR
5268.252	5268.312	-0.059	$(Hex)_2$ + $(Man)_3(GlcNAc)_2$	4050.881	184-217 NDLSYVAQYWNQTGYDLWEEVQGSSFFTIAVQHR
5430.241	5430.365	-0.123	$(Hex)_3$ + $(Man)_3(GlcNAc)_2$	4050.881	184-217 NDLSYVAQYWNQTGYDLWEEVQGSSFFTIAVQHR
5592.238	5592.417	-0.178	$(Hex)_4$ + $(Man)_3(GlcNAc)_2$	4050.881	184-217 NDLSYVAQYWNQTGYDLWEEVQGSSFFTIAVQHR
5754.240	5754.47	-0.229	$(Hex)_5$ + $(Man)_3(GlcNAc)_2$	4050.881	184-217 NDLSYVAQYWNQTGYDLWEEVQGSSFFTIAVQHR

Table S9. Glycopeptides detected using MALDI-TOF for deglycosylated form of AGAM. The numbering is for the amino acid sequence including a signal peptide.

user mass	theor. glycopeptide mass	Δmass (daltons)	glycan structure	peptide mass [M]	peptide sequence
2960.464	2960.379	0.085	$(HexNAc)_1$	2756.293	402-427 TFADGFVSIVETHAASNGSL SEQFDK
3876.860	3876.804	0.056	$(HexNAc)_1$	3672.718	402-436 TFADGFVSIVETHAASNGSL SEQFDKSSGEELSAR (1 missed cleavage)

Table S10. Glycopeptides detected using MALDI-TOF for the variant G81A (G57A in the mature enzyme without a signal peptide). The numbering is for the amino acid sequence including a signal peptide.

user mass	theor. glycopeptide mass	Δmass (daltons)	glycan structure	peptide mass [M]	peptide sequence
2960.417	2960.379	0.038	$(HexNAc)_1$	2756.293	402-427 TFADGFVSIVETHAASNGSLSEQFDK
4459.807	4459.881	-0.073	$(Hex)_5$ + $(Man)_3(GlcNAc)_2$	2756.293	402-427 TFADGFVSIVETHAASNGSLSEQFDK
3876.801	3876.804	-0.002	$(HexNAc)_1$	3672.718	402-436 TFADGFVSIVETHAASNGSLSEQFD KSSGEELSAR (1 missed cleavage)
5376.158	5376.306	-0.147	$(Hex)_5$ + $(Man)_3(GlcNAc)_2$	3672.718	402-436 TFADGFVSIVETHAASNGSLSEQFD KSSGEELSAR (1 missed cleavage)
5430.196	5430.365	-0.168	$(Hex)_3$ + $(Man)_3(GlcNAc)_2$	4050.881	184-217 NDLSYVAQYWNQTGYDLWEEVQG SSFFTIAVQHR
5592.237	5592.417	-0.179	$(Hex)_4$ + $(Man)_3(GlcNAc)_2$	4050.881	184-217 NDLSYVAQYWNQTGYDLWEEVQG SSFFTIAVQHR
5754.245	5754.470	-0.224	$(Hex)_5$ + $(Man)_3(GlcNAc)_2$	4050.881	184-217 NDLSYVAQYWNQTGYDLWEEVQG SSFFTIAVQHR

Table S11. Glycopeptides detected using MALDI-TOF for the variant D261G (D237G in the mature enzyme without a signal peptide). The numbering is for the amino acid sequence including a signal peptide.

**user mass**	**theor. glycopeptide mass**	**Δmass (daltons)**	**glycan structure**	**peptide mass [M]**	**peptide sequence**
2960.371	2960.379	-0.007	$(HexNAc)_1$	2756.293	402-427 TFADGFVSIVETHAASNGSLSEQFDK
4459.811	4459.881	-0.069	$(Hex)_5$ + $(Man)_3(GlcNAc)_2$	2756.293	402-427 TFADGFVSIVETHAASNGSLSEQFDK
5376.158	5376.306	-0.147	$(Hex)_5$ + $(Man)_3(GlcNAc)_2$	3672.718	402-436 TFADGFVSIVETHAASNGSLSE QFDKSSGEELSAR (1 missed cleavage)
5592.243	5592.417	-0.173	$(Hex)_4$ + $(Man)_3(GlcNAc)_2$	4050.881	184-217 NDLSYVAQYWNQTGYDLWEEVQG SSFFTIAVQHR
5754.261	5754.470	-0.208	$(Hex)_5$ + $(Man)_3(GlcNAc)_2$	4050.881	184-217 NDLSYVAQYWNQTGYDLWEEVQG SSFFTIAVQHR

Table S12. Glycopeptides detected using MALDI-TOF for the variant C343T (C319T in the mature enzyme without a signal peptide). The numbering is for the amino acid sequence including a signal peptide.

**user mass**	**theor. glycopeptide mass**	**Δmass (daltons)**	**glycan structure**	**peptide mass [M]**	**peptide sequence**
2960.399	2960.379	0.020	$(HexNAc)_1$	2756.293	402-427 TFADGFVSIVETHAASNGSLSEQFDK
4459.844	4459.881	-0.036	$(Hex)_5$ + $(Man)_3(GlcNAc)_2$	2756.293	402-427 TFADGFVSIVETHAASNGSLSEQFDK
5376.196	5376.306	-0.109	$(Hex)_5$ + $(Man)_3(GlcNAc)_2$	3672.718	402-436 TFADGFVSIVETHAASNGSLSEQFDK SSGEELSAR (1 missed cleavage)
5592.251	5592.417	-0.165	$(Hex)_4$ + $(Man)_3(GlcNAc)_2$	4050.881	184-217 NDLSYVAQYWNQTGYDLWEE VQGSSFFTIAVQHR

Table S13. Glycopeptides detected using MALDI-TOF for the variant C343A (C319A in the mature enzyme without a signal peptide). The numbering is for the amino acid sequence including a signal peptide.

**user mass**	**theor. glycopeptide mass**	**Δmass (daltons)**	**glycan structure**	**peptide mass [M]**	**peptide sequence**
2960.421	2960.379	0.042	$(HexNAc)_1$	2756.293	402-427 TFADGFVSIVETHAASNGSLSEQFDK
4459.801	4459.881	-0.079	$(Hex)_5$ + $(Man)_3(GlcNAc)_2$	2756.29	402-427 TFADGFVSIVETHAASNGSLSEQFDK
5376.165	5376.306	-0.140	$(Hex)_5$ + $(Man)_3(GlcNAc)_2$	3672.72	402-436 TFADGFVSIVETHAASNGSL SEQFDKSSGEELSAR (1 missed cleavage)
5430.199	5430.365	-0.165	$(Hex)_3$ + $(Man)_3(GlcNAc)_2$	4050.88	184-217 NDLSYVAQYWNQTGYDLWEE VQGSSFFTIAVQHR
5592.242	5592.417	-0.174	$(Hex)_4$ + $(Man)_3(GlcNAc)_2$	4050.88	184-217 NDLSYVAQYWNQTGYDLWEE VQGSSFFTIAVQHR
5754.273	5754.470	-0.196	$(Hex)_5$ + $(Man)_3(GlcNAc)_2$	4050.88	184-217 NDLSYVAQYWNQTGYDLWEE VQGSSFFTIAVQHR

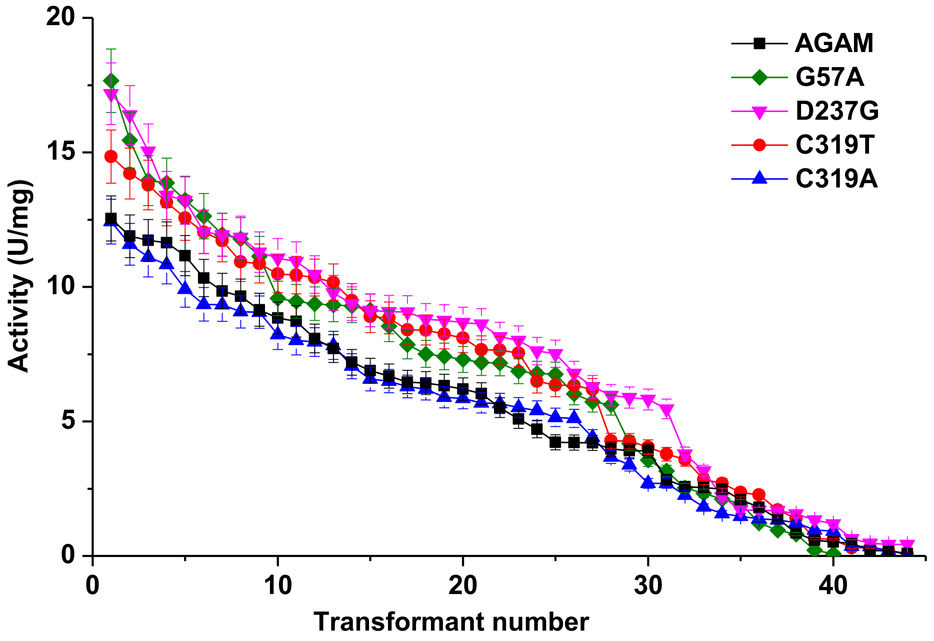

Figure S1. Enzyme activity towards soluble starch in cultures after 6 days of cultivation on plates sorted from the highest value to the lowest. Transformants that produced the variants with AA substitutions were compared to those that produced AGAM.

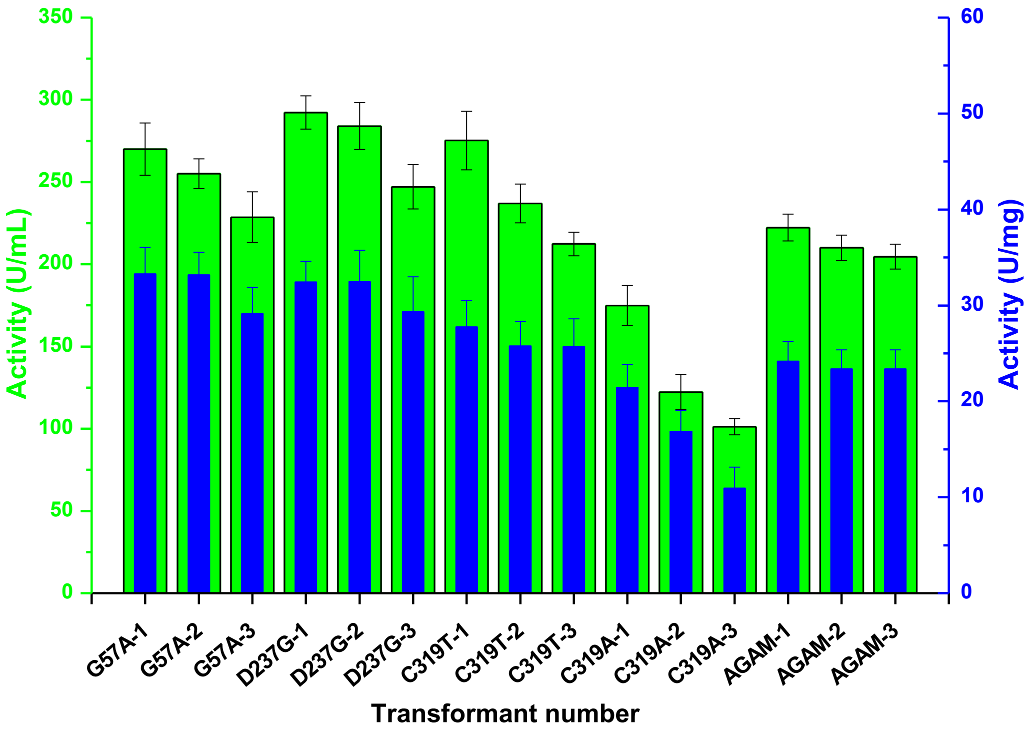

Figure S2. Enzyme activity towards soluble starch in cultures after 6 days of cultivation in flasks. Transformants that produced the variants with AA substitutions were compared to those that produced AGAM.

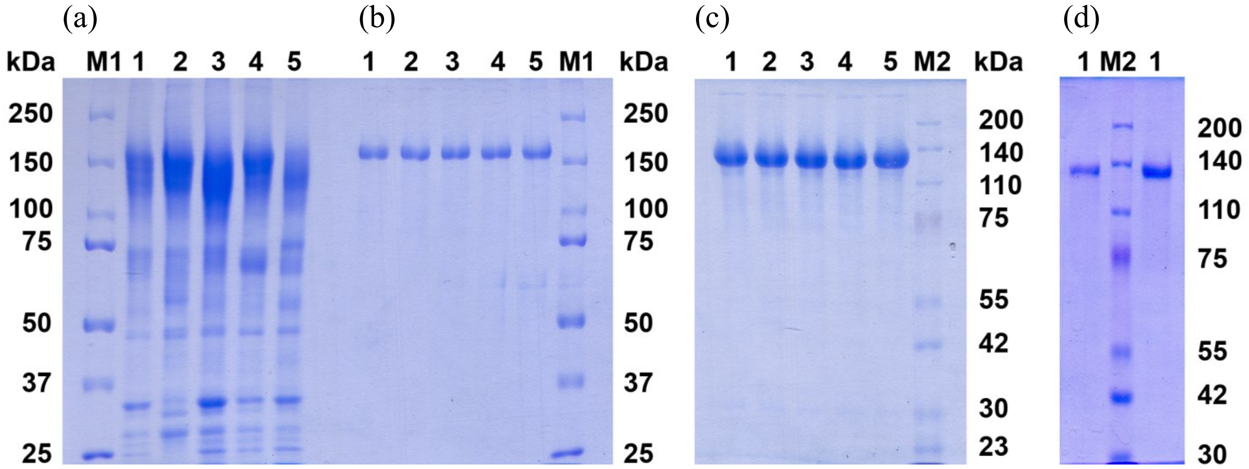

Figure S3. SDS-PAGE of crude cultures after 6 days of cultivation in flasks (a) and purified enzymes (b–d) for AGAM and the variants G57A, D237G, C319T, and C319A (1, 2, 3, 4, and 5, respectively). The SDS-PAGE was performed on PAAG gels with different compositions, 8%T (a, b, and c) and 6%T (d), and different sample dosages (b, c, and d). Prior to electrophoresis, the samples were treated with 1% SDS and 5% 2-ME at 100 °C for 20 min. M1 and M2 correspond to protein markers with molecular masses in kDa.

```
      1(-24)                          25(1)                       50(26)
AGAM  MSFRSLLALS GLVCSGLASV ISKRATLDSW LSNEATVART AILNNIGADG
dAGAM MSFRSLLALS GLVCSGLASV ISKRATLDSW LSNEATVART AILNNIGADG
G57A  MSFRSLLALS GLVCSGLASV ISKRATLDSW LSNEATVART AILNNIGADG
D237G MSFRSLLALS GLVCSGLASV ISKRATLDSW LSNEATVART AILNNIGADG
C319T MSFRSLLALS GLVCSGLASV ISKRATLDSW LSNEATVART AILNNIGADG
C319A MSFRSLLALS GLVCSGLASV ISKRATLDSW LSNEATVART AILNNIGADG

      51(27)                          81(57)                 100(76)
AGAM  AWVSGADSGI VVASPSTDNP DYFYTWTRDS GLVIKSLVDL FRNGDTSLLS
dAGAM AWVSGADSGI VVASPSTDNP DYFYTWTRDS GLVIKSLVDL FRNGDTSLLS
G57A  AWVSGADSGI VVASPSTDNP DYFYTWTRDS ALVIKSLVDL FRNGDTSLLS
D237G AWVSGADSGI VVASPSTDNP DYFYTWTRDS GLVIKSLVDL FRNGDTSLLS
C319T AWVSGADSGI VVASPSTDNP DYFYTWTRDS GLVIKSLVDL FRNGDTSLLS
C319A AWVSGADSGI VVASPSTDNP DYFYTWTRDS GLVIKSLVDL FRNGDTSLLS

      101(77)                                                150(126)
AGAM  TIEHYISSQA IIQGVSNPSG DLSSGGLGEP KFNVDETAYT GSWGRPQRDG
dAGAM TIEHYISSQA IIQGVSNPSG DLSSGGLGEP KFNVDETAYT GSWGRPQRDG
G57A  TIEHYISSQA IIQGVSNPSG DLSSGGLGEP KFNVDETAYT GSWGRPQRDG
D237G TIEHYISSQA IIQGVSNPSG DLSSGGLGEP KFNVDETAYT GSWGRPQRDG
C319T TIEHYISSQA IIQGVSNPSG DLSSGGLGEP KFNVDETAYT GSWGRPQRDG
C319A TIEHYISSQA IIQGVSNPSG DLSSGGLGEP KFNVDETAYT GSWGRPQRDG

      151(127)                                               200(176)
                                                         194(170)
AGAM  PALRATAMID FGNWLIDNGY TSVATDIVWP LVRNDLSYVA QYWNQTGYDL
dAGAM PALRATAMID FGNWLIDNGY TSVATDIVWP LVRNDLSYVA QYWNQTGYDL
G57A  PALRATAMID FGNWLIDNGY TSVATDIVWP LVRNDLSYVA QYWNQTGYDL
D237G PALRATAMID FGNWLIDNGY TSVATDIVWP LVRNDLSYVA QYWNQTGYDL
C319T PALRATAMID FGNWLIDNGY TSVATDIVWP LVRNDLSYVA QYWNQTGYDL
C319A PALRATAMID FGNWLIDNGY TSVATDIVWP LVRNDLSYVA QYWNQTGYDL

      201(177)                          233(209)             250(226)
          205(181)                         236(212)  245(221)
AGAM  WEEVQGSSFF TIAVQHRALV EGSAFATAVG SSCSWCDSQA PQILCYLQSF
dAGAM WEEVQGSSFF TIAVQHRALV EGSAFATAVG SSCSWCDSQA PQILCYLQSF
G57A  WEEVQGSSFF TIAVQHRALV EGSAFATAVG SSCSWCDSQA PQILCYLQSF
D237G WEEVQGSSFF TIAVQHRALV EGSAFATAVG SSCSWCDSQA PQILCYLQSF
C319T WEEVQGSSFF TIAVQHRALV EGSAFATAVG SSCSWCDSQA PQILCYLQSF
C319A WEEVQGSSFF TIAVQHRALV EGSAFATAVG SSCSWCDSQA PQILCYLQSF

      251(227)                                               300(276)
                 261(237)                 285(261)  293(269)
AGAM  WTGSYILANF DSSRSGKDAN TLLGSIHTFD PEAGCDDSTF QPCSPRALAN
dAGAM WTGSYILANF DSSRSGKDAN TLLGSIHTFD PEAGCDDSTF QPCSPRALAN
G57A  WTGSYILANF DSSRSGKDAN TLLGSIHTFD PEAGCDDSTF QPCSPRALAN
D237G WTGSYILANF GSSRSGKDAN TLLGSIHTFD PEAGCDDSTF QPCSPRALAN
C319T WTGSYILANF DSSRSGKDAN TLLGSIHTFD PEAGCDDSTF QPCSPRALAN
C319A WTGSYILANF DSSRSGKDAN TLLGSIHTFD PEAGCDDSTF QPCSPRALAN

      301(277)                                               350(326)
                                                        343(319)
AGAM  HKEVVDSFRS IYTINDGLSD SEAVAVGRYP EDSYYNGNPW FLCTLAAAEQ
dAGAM HKEVVDSFRS IYTINDGLSD SEAVAVGRYP EDSYYNGNPW FLCTLAAAEQ
G57A  HKEVVDSFRS IYTINDGLSD SEAVAVGRYP EDSYYNGNPW FLCTLAAAEQ
D237G HKEVVDSFRS IYTINDGLSD SEAVAVGRYP EDSYYNGNPW FLCTLAAAEQ
C319T HKEVVDSFRS IYTINDGLSD SEAVAVGRYP EDSYYNGNPW FLTTLAAAEQ
C319A HKEVVDSFRS IYTINDGLSD SEAVAVGRYP EDSYYNGNPW FLATLAAAEQ
```

```
      351(327)                                           400(376)
AGAM  LYDALYQWDK QGELEITDVS LDFFQALYSS AATGTYSSSS STYSSIVDAV
dAGAM LYDALYQWDK QGELEITDVS LDFFQALYSS AATGTYSSSS STYSSIVDAV
G57A  LYDALYQWDK QGELEITDVS LDFFQALYSS AATGTYSSSS STYSSIVDAV
D237G LYDALYQWDK QGELEITDVS LDFFQALYSS AATGTYSSSS STYSSIVDAV
C319T LYDALYQWDK QGELEITDVS LDFFQALYSS AATGTYSSSS STYSSIVDAV
C319A LYDALYQWDK QGELEITDVS LDFFQALYSS AATGTYSSSS STYSSIVDAV

      401(377)          418(394)                         450(426)
AGAM  KTFADGFVSI VETHAASNGS LSEQFDKSSG EELSARDLTW SYAALLTANN
dAGAM KTFADGFVSI VETHAASNGS LSEQFDKSSG EELSARDLTW SYAALLTANN
G57A  KTFADGFVSI VETHAASNGS LSEQFDKSSG EELSARDLTW SYAALLTANN
D237G KTFADGFVSI VETHAASNGS LSEQFDKSSG EELSARDLTW SYAALLTANN
C319T KTFADGFVSI VETHAASNGS LSEQFDKSSG EELSARDLTW SYAALLTANN
C319A KTFADGFVSI VETHAASNGS LSEQFDKSSG EELSARDLTW SYAALLTANN

      451(427)              472(448)                     500(476)
AGAM  RRNSVVPPSW GETSASSVPG SCVATSASGT YSSVTVTSWP SIVATGGTTT
dAGAM RRNSVVPPSW GETSASSVPG SCVATSASGT YSSVTVTSWP SIVATGGTTT
G57A  RRNSVVPPSW GETSASSVPG SCVATSASGT YSSVTVTSWP SIVATGGTTT
D237G RRNSVVPPSW GETSASSVPG SCVATSASGT YSSVTVTSWP SIVATGGTTT
C319T RRNSVVPPSW GETSASSVPG SCVATSASGT YSSVTVTSWP SIVATGGTTT
C319A RRNSVVPPSW GETSASSVPG SCVATSASGT YSSVTVTSWP SIVATGGTTT

      501(477)                          533(509)         550(526)
AGAM  TTATTTGSGG VTSTSKTTTT ASKTSTTTSS TSCTTPTAVA VTFDLTATTT
dAGAM TTATTTGSGG VTSTSKTTTT ASKTSTTTSS TSCTTPTAVA VTFDLTATTT
G57A  TTATTTGSGG VTSTSKTTTT ASKTSTTTSS TSCTTPTAVA VTFDLTATTT
D237G TTATTTGSGG VTSTSKTTTT ASKTSTTTSS TSCTTPTAVA VTFDLTATTT
C319T TTATTTGSGG VTSTSKTTTT ASKTSTTTSS TSCTTPTAVA VTFDLTATTT
C319A TTATTTGSGG VTSTSKTTTT ASKTSTTTSS TSCTTPTAVA VTFDLTATTT

      551(527)                                           600(576)
AGAM  YGESIYLVGS ISQLGDWDTS DGVALSADKY TSSNPLWYVT VTLPAGESFE
dAGAM YGESIYLVGS ISQLGDWDTS DGVALSADKY TSSNPLWYVT VTLPAGESFE
G57A  YGESIYLVGS ISQLGDWDTS DGVALSADKY TSSNPLWYVT VTLPAGESFE
D237G YGESIYLVGS ISQLGDWDTS DGVALSADKY TSSNPLWYVT VTLPAGESFE
C319T YGESIYLVGS ISQLGDWDTS DGVALSADKY TSSNPLWYVT VTLPAGESFE
C319A YGESIYLVGS ISQLGDWDTS DGVALSADKY TSSNPLWYVT VTLPAGESFE

      601(577)                     628(604)     640(616)
AGAM  YKFIRVESDD TVEWESDPNR EYTVPQTCGE STVTVTDTWR
dAGAM YKFIRVESDD TVEWESDPNR EYTVPQTCGE STVTVTDTWR
G57A  YKFIRVESDD TVEWESDPNR EYTVPQTCGE STVTVTDTWR
D237G YKFIRVESDD TVEWESDPNR EYTVPQTCGE STVTVTDTWR
C319T YKFIRVESDD TVEWESDPNR EYTVPQTCGE STVTVTDTWR
C319A YKFIRVESDD TVEWESDPNR EYTVPQTCGE STVTVTDTWR
```

Figure S4. Peptides were detected using MALDI-TOF in the AA sequences of AGAM, dAGAM (after deglycosylation with Endo F1), and the variants. The signal peptide is shown in italics and bold. The detected peptides are shown in green font, while those undetected are in black font. Cysteine residues, sites of *N*-glycosylation, and sites of AA substitutions are shown in yellow, grey, and cyan, respectively. Acrylamide-modified cysteine residues (Cys_PAM) are underlined, and unmodified residues are in strikethrough. Oxidized tryptophan (TPO) is underlined and reduced tryptophan is in strikethrough, while oxidized methionine (MSO) is also underlined and reduced methionine is in strikethrough. The numbering of the mature enzyme without the signal peptide is given in round brackets.

(a) 265(241)-296(272) peptide
(R)/SGKDANTLLGSIHTFDPEAG<u>C</u>DDSTFQP<u>C</u>SPR/(A)

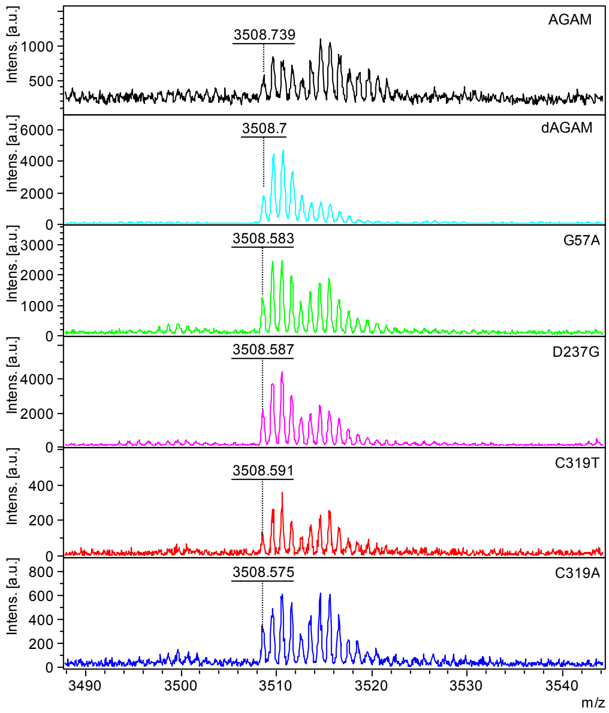

(b) 268(244)-296(272) peptide
(K)/DANTLLGSIHTFDPEAG<u>C</u>DDSTFQP~~C~~SPR/(A)
or
(K)/DANTLLGSIHTFDPEAG~~C~~DDSTFQP<u>C</u>SPR/(A)

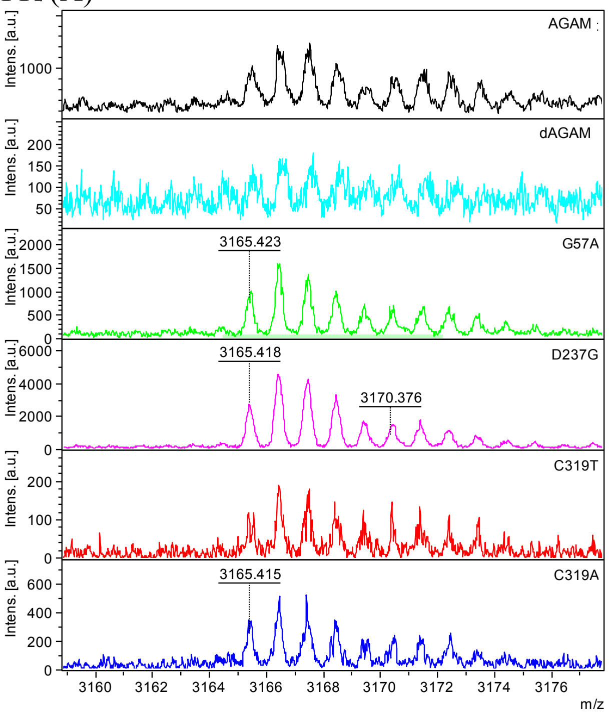

(c) 621(597)-640(616) peptide
(R)/EYTVPQT~~C~~GESTVTVTDTWR

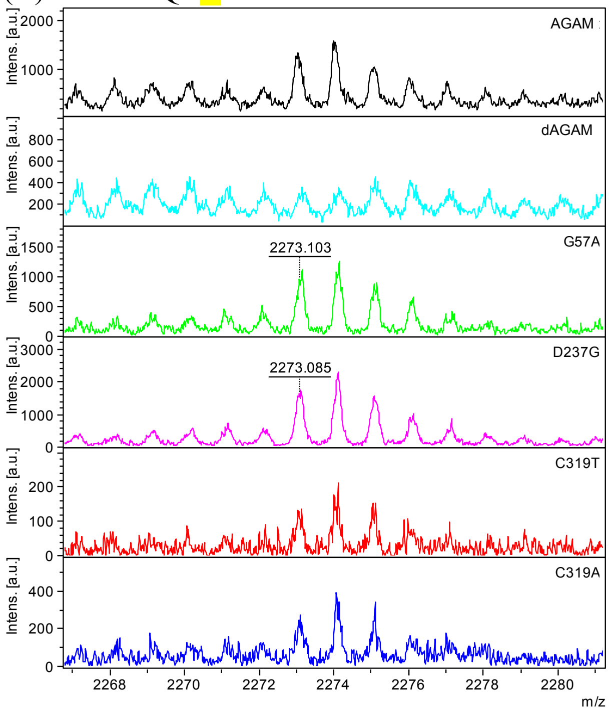

(d) 621(597)-640(616) peptide
(R)/EYTVPQT<u>C</u>GESTVTVTDTWR

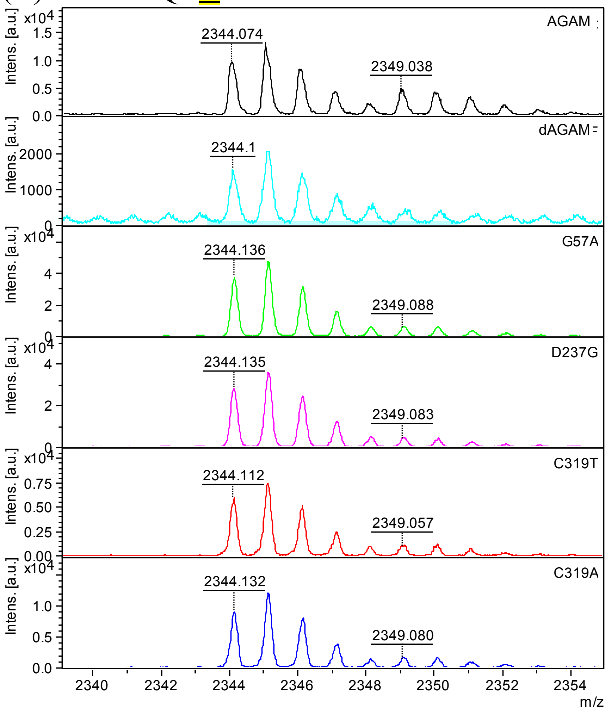

Figure S5. Peaks were detected using MALDI-TOF for peptides containing cysteine residues in the AA sequences of AGAM, dAGAM (after treatment with Endo F1), and the variants. The cysteine residues are shown in yellow. Acrylamide-modified cysteine residues (Cys_PAM) are underlined, and unmodified residues are in strikethrough. The numbering of the mature enzyme without the signal peptide is given in round brackets.

Peptide mass	Position	#MC	Artif.modification(s)		Peptide sequence	Comments
5037.3168	218-264	0	Cys_PAM: 233, 236, 245	5250.4281	ALVEGSAFATAVGSSC SWCDSQAPQILCYLQS FWTGSYILANFDSSR	Not found
5309.4652	218-267	1	Cys_PAM: 233, 236, 245	5522.5766	ALVEGSAFATAVGSSC SWCDSQAPQILCYLQS FWTGSYILANFDSSRSG K	Not found
3366.5001	265-296	1	Cys_PAM: 285, 293	3508.5743	SGKDANTLLGSIHTFDP EAGCDDSTFQPCSPR	Cys_PAM(285, 293) detected in all samples (Figure S5 a)
4000.8552	265-302	2	Cys_PAM: 285, 293	4142.9294	SGKDANTLLGSIHTFDP EAGCDDSTFQPCSPRA LANHK	Not found
4833.2631	265-309	3	Cys_PAM: 285, 293	4975.3373	SGKDANTLLGSIHTFDP EAGCDDSTFQPCSPRA LANHKEVVDSFR	Not found
3094.3516	268-296	0	Cys_PAM: 285, 293	3236.4259	DANTLLGSIHTFDPEAG CDDSTFQPCSPR	Cys_PAM(285 or 293) detected in several samples (Figure S5 b)
3728.7067	268-302	1	Cys_PAM: 285, 293	3870.7810	DANTLLGSIHTFDPEAG CDDSTFQPCSPRALAN HK	Not found
4561.1146	268-309	2	Cys_PAM: 285, 293	4703.1889	DANTLLGSIHTFDPEAG CDDSTFQPCSPRALAN HKEVVDSFR	Not found
5795.6798	310-360	1	Cys_PAM: 343	5866.7169	SIYTINDGLSDSEAVAV GRYPEDSYYNGNPWFL CTLAAAEQLYDALYQ WDK	Not found
3847.7260	329-360	0	Cys_PAM: 343	3918.7632	YPEDSYYNGNPWFLCT LAAAEQLYDALYQWD K	Similar to peak with m/z 3847.9475 for peptide 149-183
6211.9735	452-516	1	Cys_PAM: 472	6283.0106	RNSVVPPSWGETSASS VPGSCVATSASGTYSS VTVTSWPSIVATGGTT TTTATTTGSGGVTSTSK	Not found
6055.8724	453-516	0	Cys_PAM: 472	6126.9095	NSVVPPSWGETSASSV PGSCVATSASGTYSSVT VTSWPSIVATGGTTTTT ATTTGSGGVT STSK	Not found
6385.0450	517-579	1	Cys_PAM: 533	6456.0821	TTTTASKTSTTTSSTSC TTPTAVAVTFDLTATT TYGESIYLVGSISQLGD WDTSDGVALSADK	Not found
5694.6902	524-579	0	Cys_PAM: 533	5765.7273	TSTTTSSTSCTTPTAVA VTFDLTATTTYGESIYL VGSISQLGDWDTSDGV ALSADK	Not found
4031.7723	606-640	1	Cys_PAM: 628	4102.8094	VESDDTVEWESDPNRE YTVPQTCGESTVTVTD TWR	Not found
2273.0390	621-640	0	Cys_PAM: 628	2344.0761	EYTVPQTCGESTVTVT DTWR	Unmodified detected in several samples (Figure S5 c), Cys_PAM(628) detected in all samples (Figure S5 d)

Figure S6. Theoretical peptides resulting from the cleavage of AGAM by trypsin in the mass range of m/z 500–6500. The numbering is for the amino acid sequence including a signal peptide.

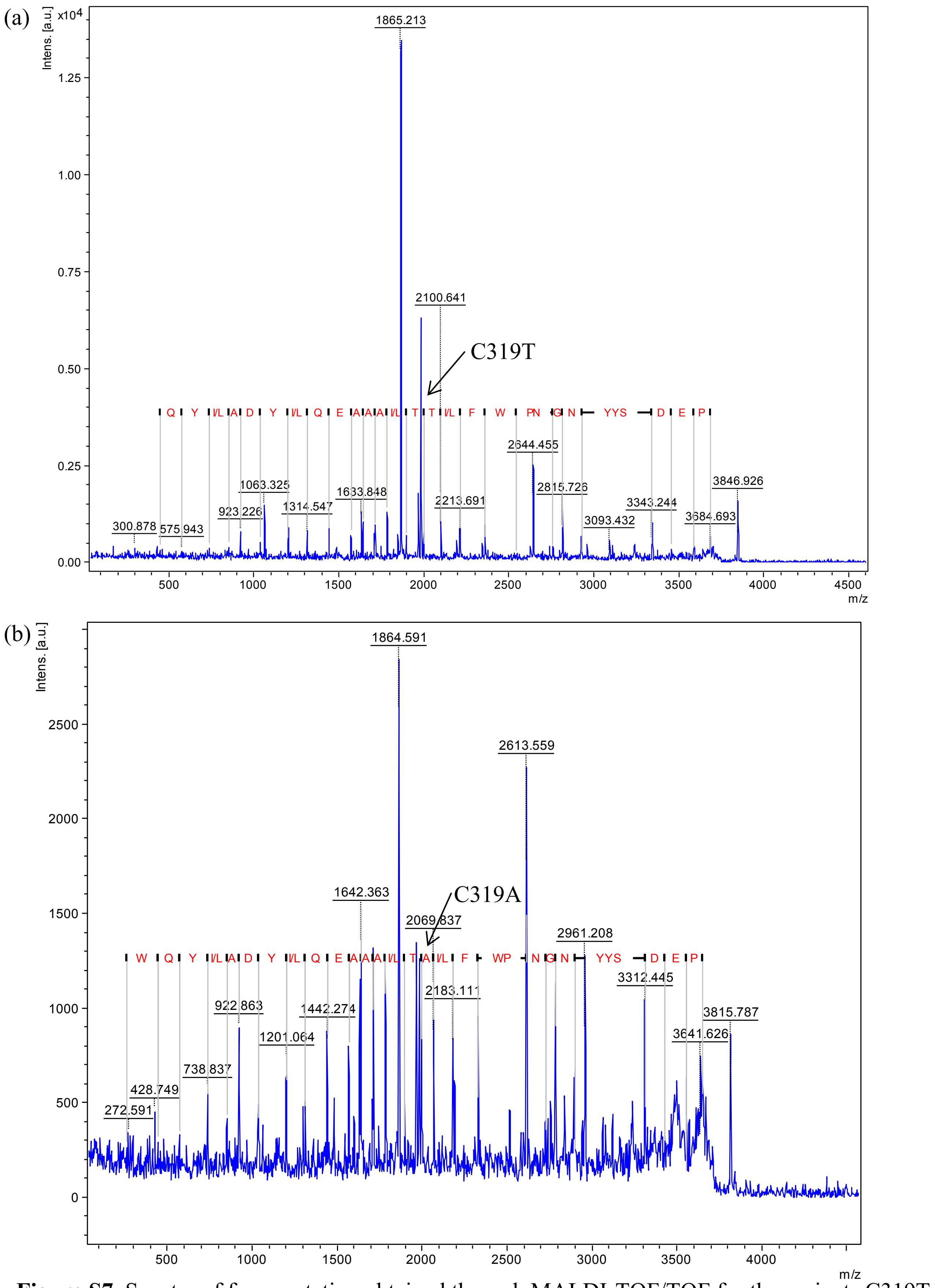

Figure S7. Spectra of fragmentation obtained through MALDI-TOF/TOF for the variants C319T (a) and C319A (b).

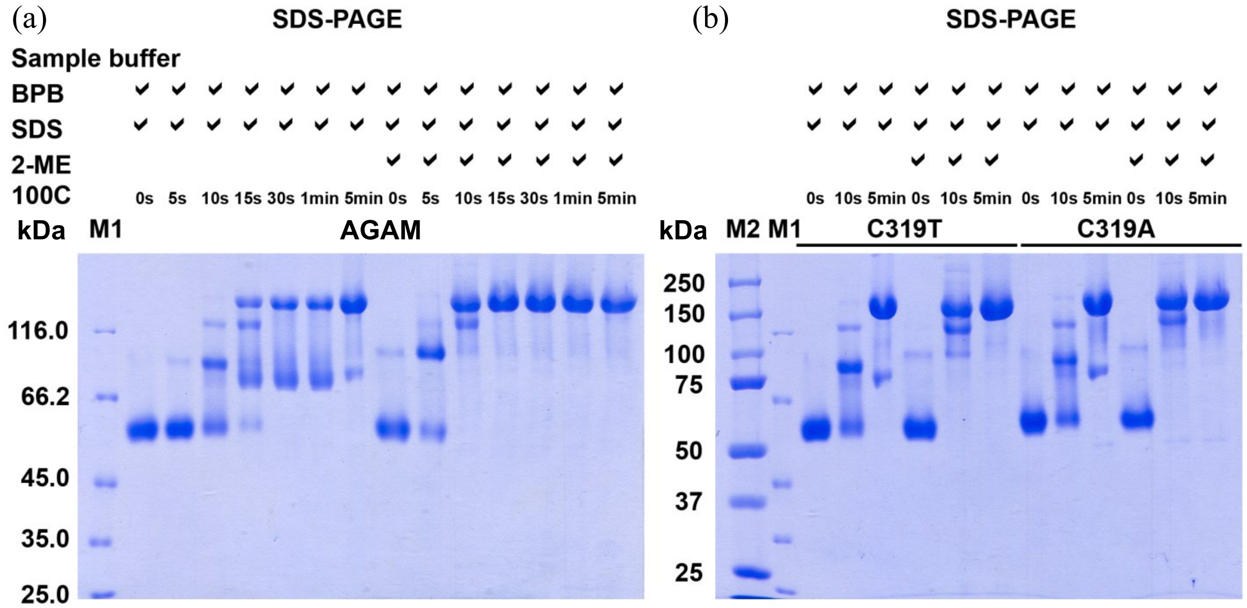

Figure S8. SDS-PAGE of AGAM (a) and the variants C319T and C319A (b). The samples were treated with a sample buffer containing 0.05% BPB, 1% SDS, or 5% 2-ME at 100 °C for 0 s – 5 min. SDS-PAGE was performed on PAAG gels with 8%T. The M1 and M2 lanes correspond to protein markers with molecular masses in kDa.

(a) $(HexNAc)_1$ linked to peptide 184(160)–217(193)

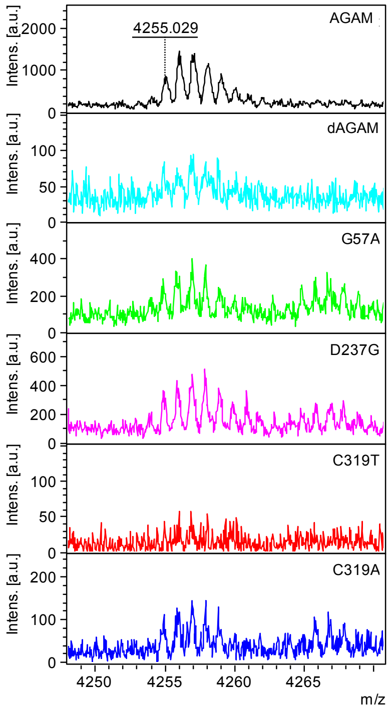

(b) $(Hex)_2$+$(Man)_3(GlcNAc)_2$ linked to peptide 184(160)–217(193)

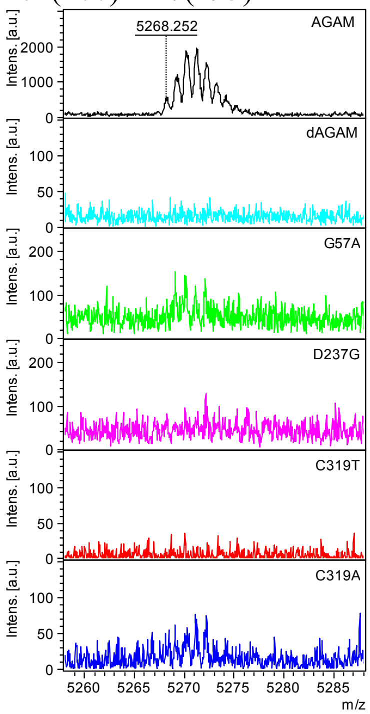

(c) $(Hex)_{3\text{–}5}$+$(Man)_3(GlcNAc)_2$ linked to peptide 184(160)–217(193)

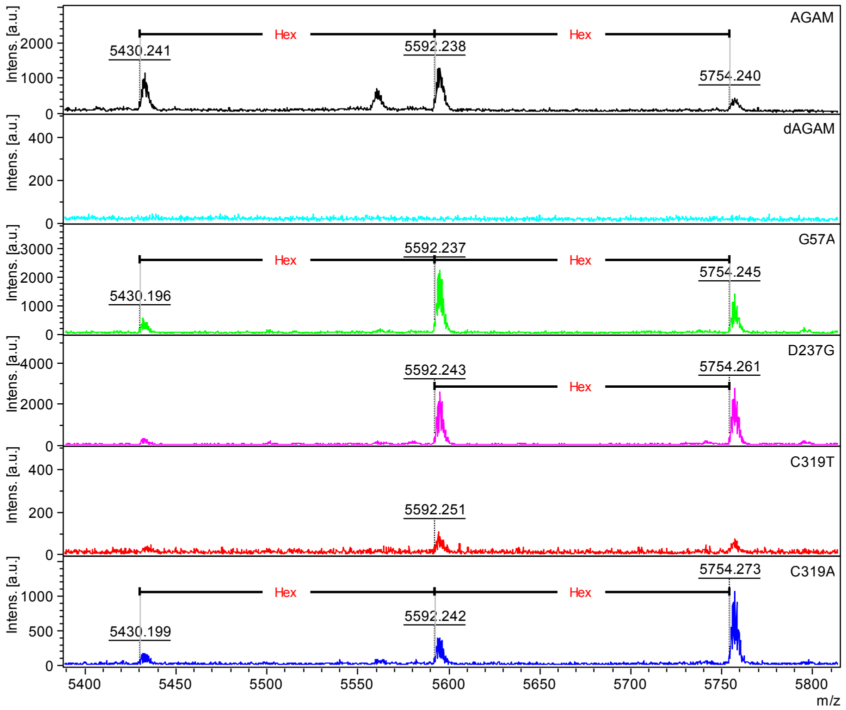

Figure S9. Glycosylation linked to the site N170 in AGAM, dAGAM (after treatment with Endo F1), and the variants. A peak of $(HexNAc)_1$+peptide was detected with a significant intensity in AGAM but a low intensity in dAGAM and the variants (a). A peak of $(Hex)_2$+$(Man)_3(GlcNAc)_2$+peptide was detected in AGAM but not in other forms (b). A series of of high-mannose peaks $(Hex)_{3\text{–}5}$+$(Man)_3(GlcNAc)_2$+peptide were detected in AGAM and the variants, but not in dAGAM (c). The numbering of the mature enzyme without the signal peptide is given in round brackets.

(a) $(HexNAc)_1$ (b) $(Hex)_2+(Man)_3(GlcNAc)_2$ (c) $(Hex)_5+(Man)_3(GlcNAc)_2$

glycans linked to peptide 402(378)–427(403)

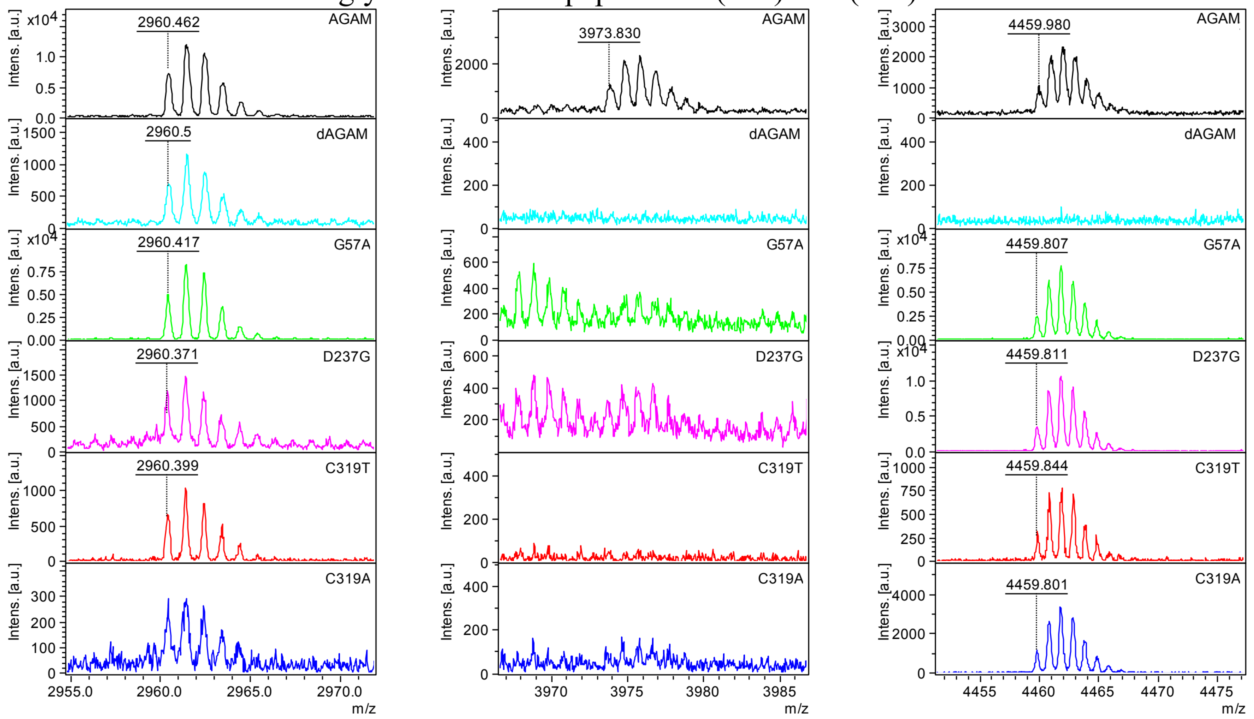

(d) $(HexNAc)_1$ (e) $(Hex)_2+(Man)_3(GlcNAc)_2$ (f) $(Hex)_5+(Man)_3(GlcNAc)_2$

glycans linked to peptide 402(378)–436(412)

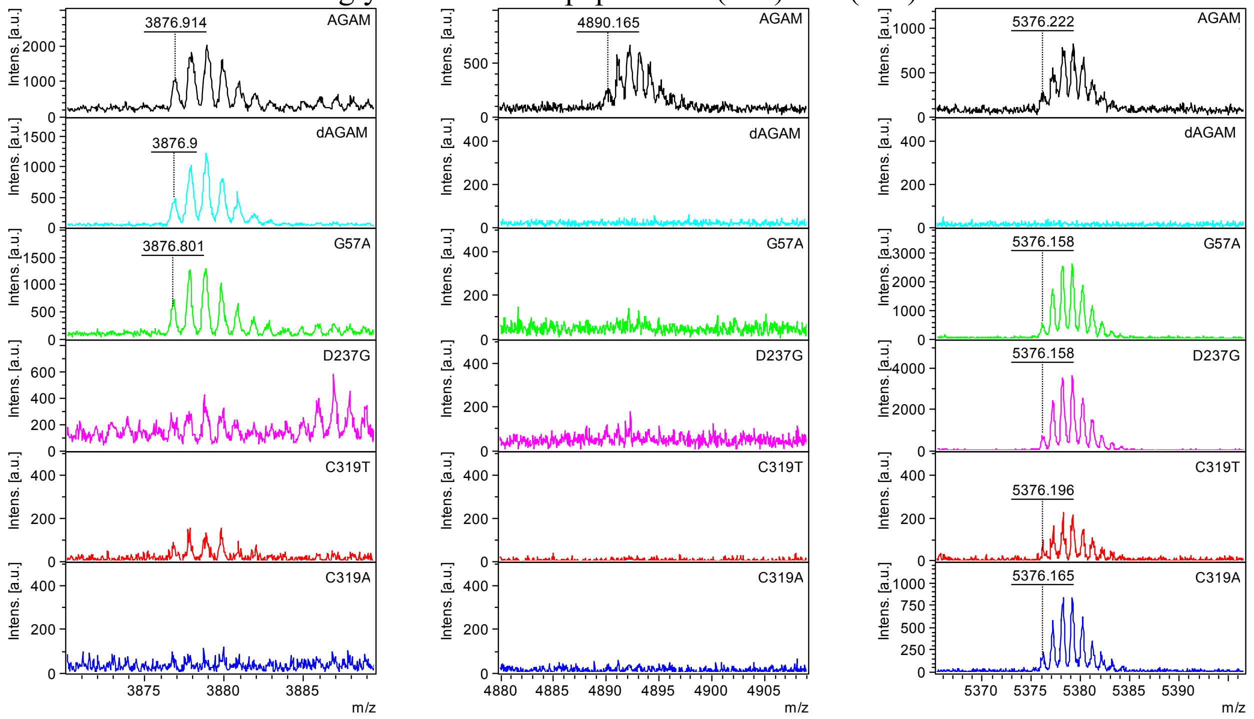

Figure S10. Glycosylation linked to the site N394 in AGAM, dAGAM (after treatment with Endo F1), and the variants. A peak of $(HexNAc)_1$+peptide was detected with a significant intensity in all forms of the enzyme for the peptide 402(378)–427(403) (a) and some forms for the peptide 402(378)–436(412) (d). A peak of $(Hex)_2+(Man)_3(GlcNAc)_2$+peptide was detected with a significant intensity in AGAM for two peptides, but not found in other forms (b and e). A high-mannose peak $(Hex)_5+(Man)_3(GlcNAc)_2$+peptide was detected in AGAM and the variants, but not in dAGAM (c and f). The numbering of the mature enzyme without the signal peptide is given in round brackets.

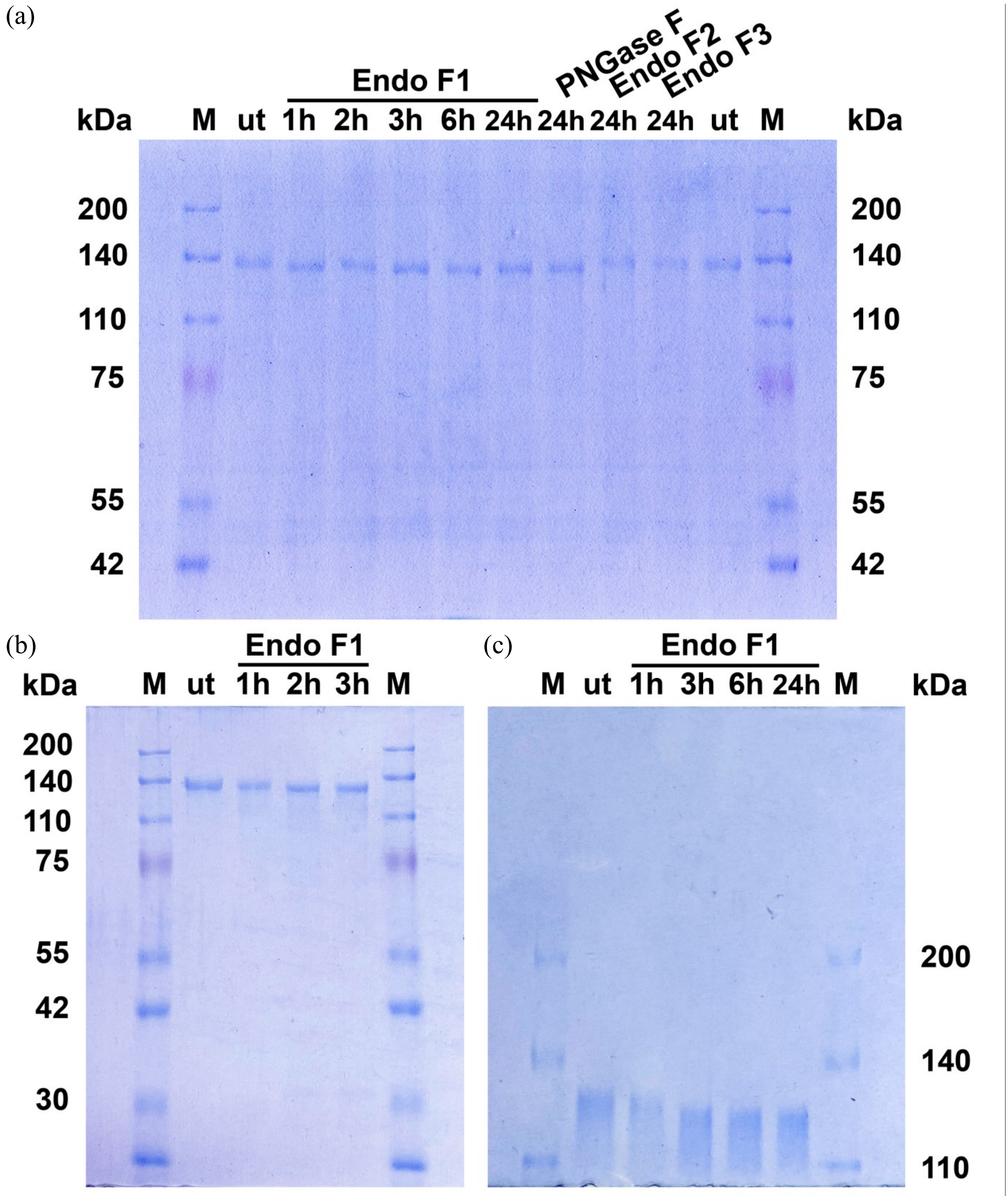

Figure S11. SDS-PAGE of AGAM treated with Endo F1, Endo F2, and Endo F3, as well as PNGase F. Untreated samples are shown as "ut", and the duration of treatment was 1–24 h. SDS-PAGE was performed on PAAG gels with 6%T (a), 8%T (b), and 4%T (c). The M lanes on the side correspond to protein marker with molecular masses in kDa.

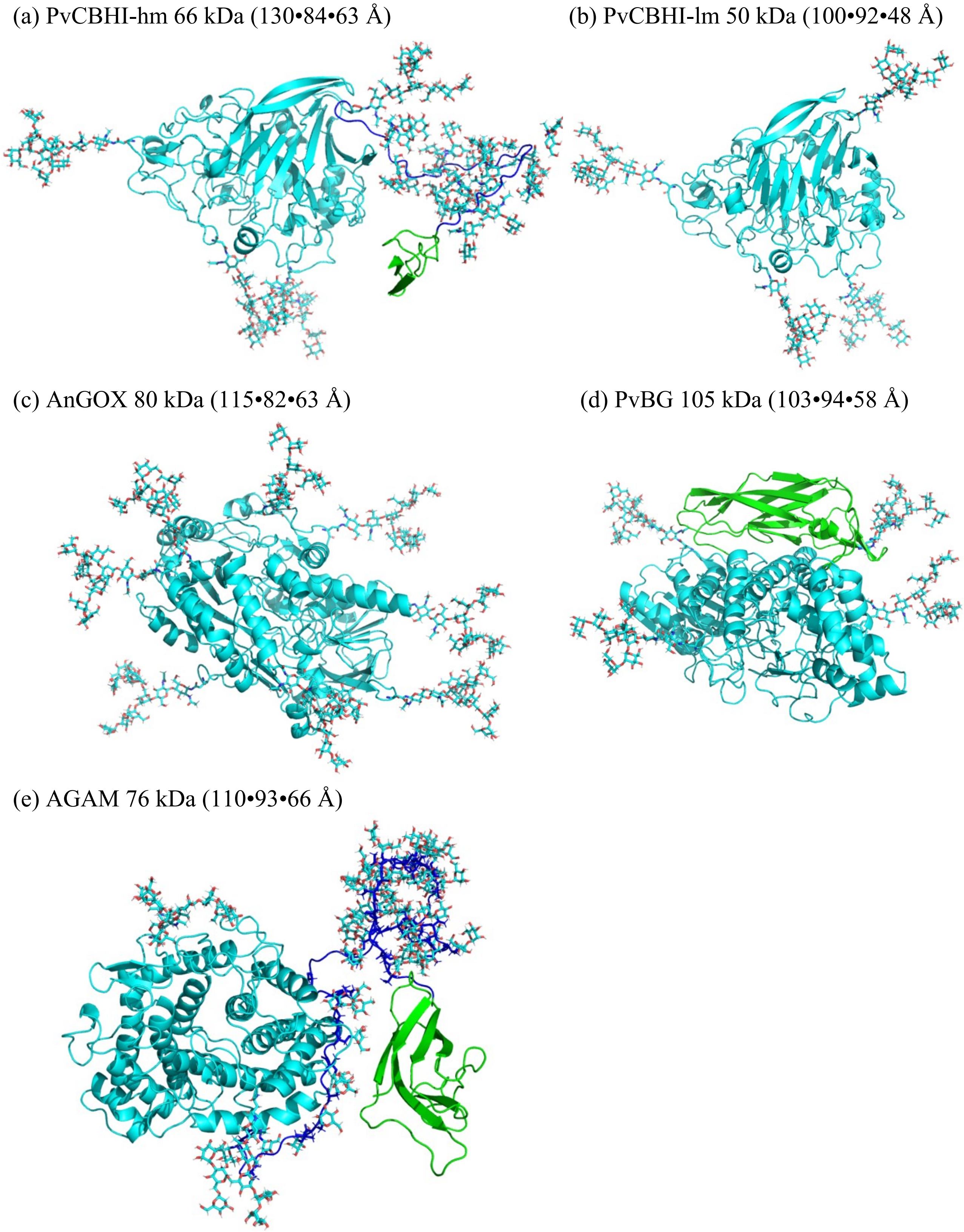

Figure S12. Spatial structures of *P. verruculosum* cellobiohydrolase I in full-length (a) and truncated (b) forms (PvCBHI-hm and PvCBHI-lm, respectively), *A. niger* glucose oxidase (AnGOX) (c), *P. verruculosum* beta-glucosidase (PvBG) (d), and AGAM (e). A catalytic domain, a linker, and a substrate-binding domain in PvCBHI-hm and AGAM are shown in cyan, blue, and green, respectively. The two structural domains in PvBG (d) are shown in cyan and green. Glycans are shown as sticks. The spatial dimensions of the structures were calculated using VMD 1.9.4.

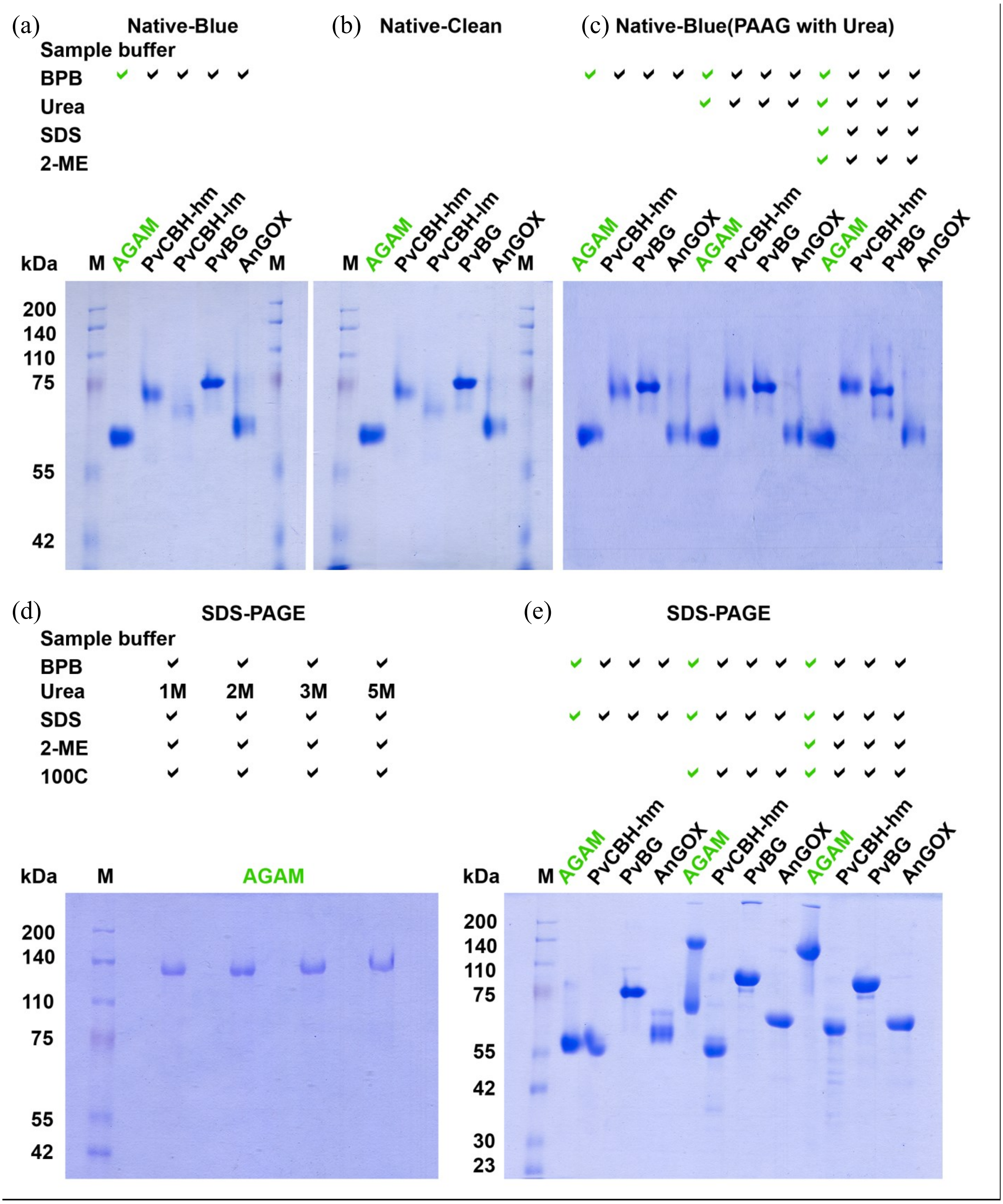

Figure S13. Native-Blue (a), Native-Clean (b), Native-Blue (with 0.015 M urea in PAAG) (c), and SDS-PAGE (d and e) of AGAM, *P. verruculosum* cellobiohydrolase I in full-length and truncated forms (PvCBHI-hm and PvCBHI-lm, respectively), *P. verruculosum* beta-glucosidase (PvBG), and *A. niger* glucose oxidase (AnGOX). The samples were treated with a sample buffer containing 0.05% BPB, 1% SDS, 5% 2-ME, or urea (0.015 M in c and 1–5 M in d) at room temperature or at 100 °C for 5 min. SDS-PAGE was performed on PAAG gels with 6%T (a–d) and 8%T (e). The M lanes on the side correspond to protein marker with molecular masses in kDa.

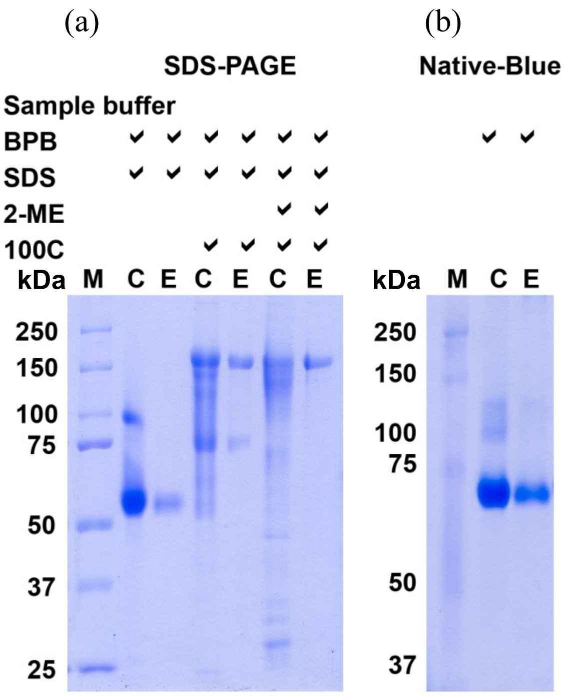

Figure S14. SDS-PAGE (a) and Native-Blue (b) of a crude flask solution (marked as "C") and a purified enzyme (marked as "E") of AGAM. The samples were treated with a sample buffer containing 0.05% BPB, 1% SDS, or 5% 2-ME at room temperature or at 100 °C for 5 min. SDS-PAGE and Native-Blue were performed on PAAG gels with 8%T and 6%T, respectively. The M lanes on the side correspond to protein marker with molecular masses in kDa.

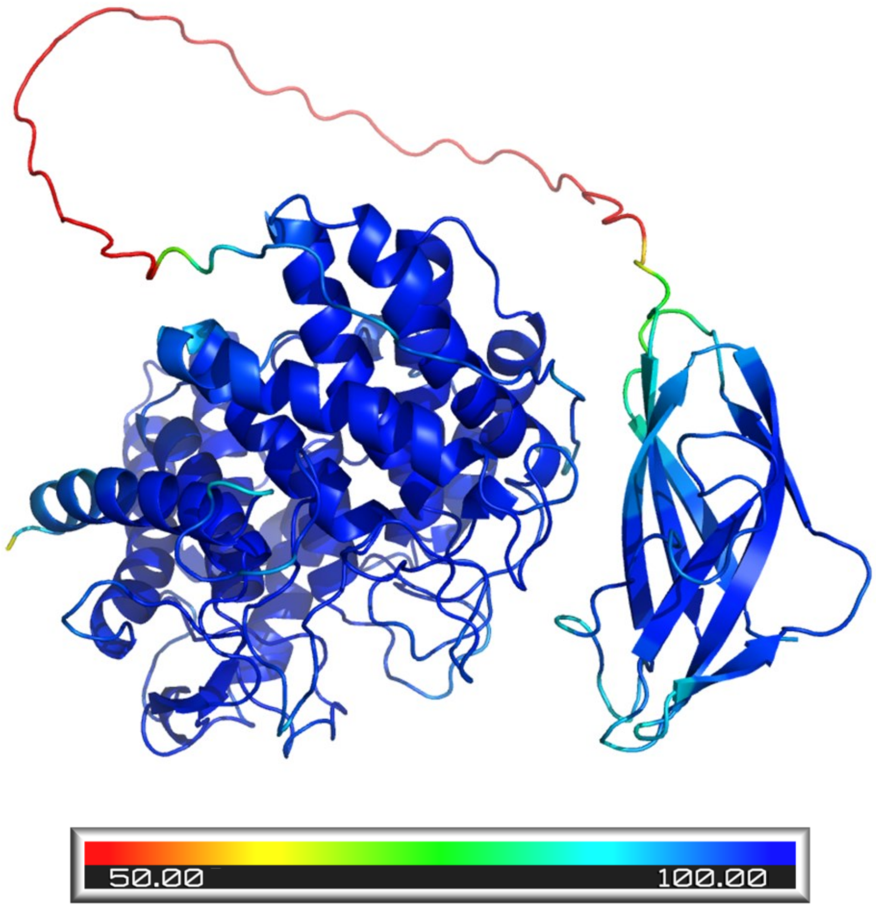

Figure S15. A full-length model AGAM constructed using AlphaFold 2.0, with an accuracy of pLDDT=92.6 and pTM=0.772. Residues 3–441 in the catalytic domain were predicted with pLDDT values ranging from 88.50 to 98.94. Residues 517–616 in the substrate-binding domain were predicted with pLDDT values ranging from 73.69 to 98.56. Residues in the linker region had lower pLDDT values: 40.81–98.12 for residues 442–473, 22.53–35.66 for residues 474–506, and 45.53–90.06 for residues 507–516.

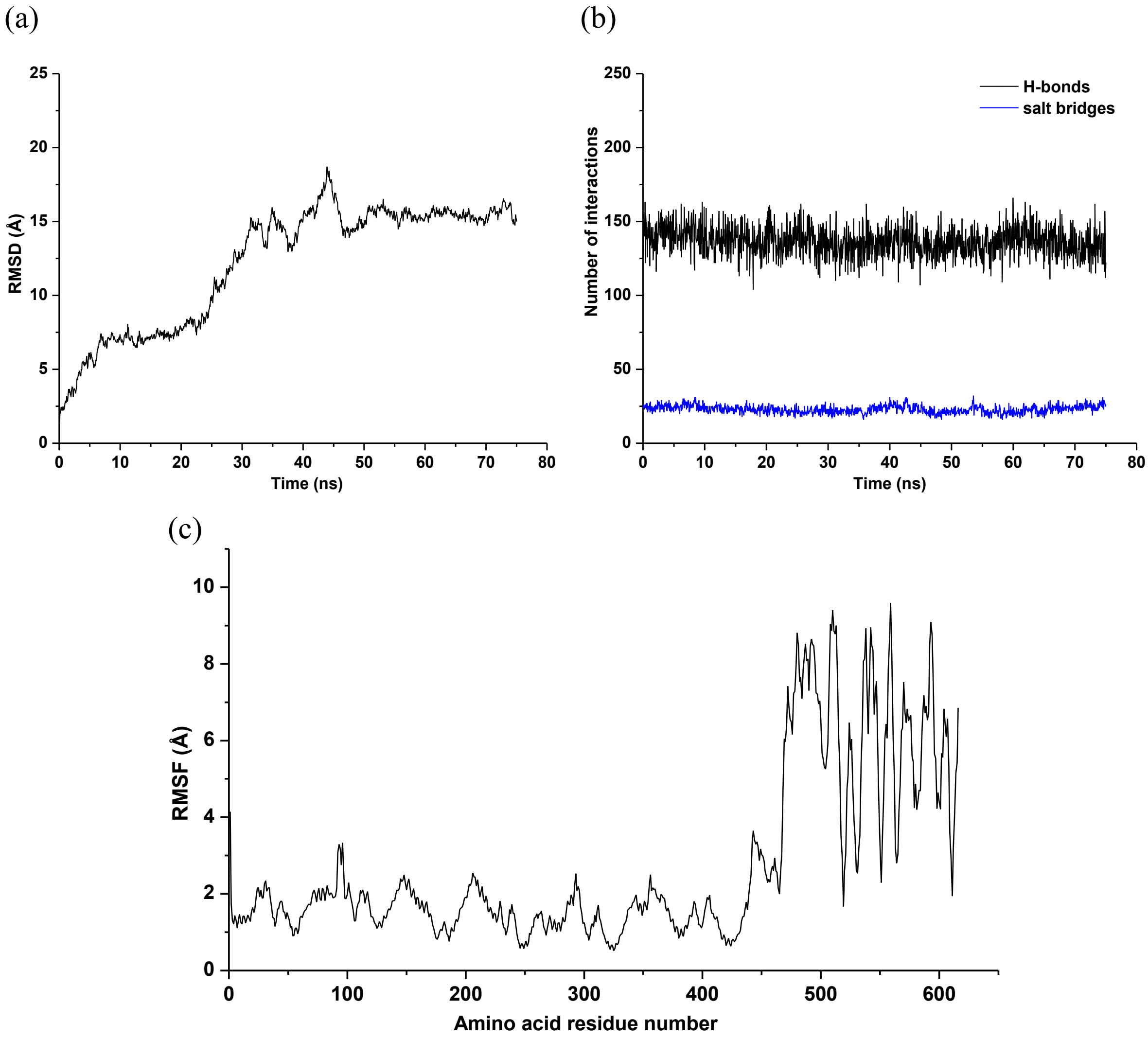

Figure S16. MD simulations of AGAM as a monomer and glycoprotein at 30 °C for 75 ns. The model of AGAM was created using AlphaFold 2.0, and glycans were added using Glycan Reader and Modeler. *N*-linked glycans were $(Man)_8(GlcNAc)_2$ at glycosylation sites N170 and N394; *O*-linked glycans were $(Man)_{1\text{-}2}$ in the linker sequence. RMSD (a), number of H-bonds and salt bridges (b), and RMSF (c) were calculated for amino acid residues. The time interval from 50 to 75 ns was used to calculate RMSF (c). Amino acid residues 1–441, 442–508, and 509–616 correspond to the catalytic domain, linker, and substrate-binding domain, respectively. The numbering was for the mature enzyme without a signal peptide.

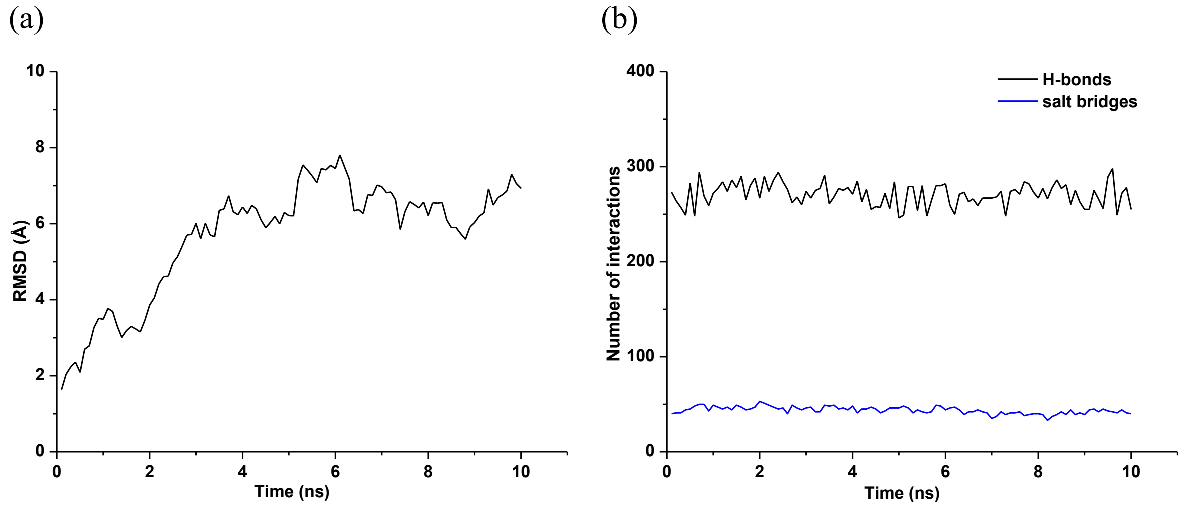

Figure S17. MD simulations of AGAM as a dimer and glycoprotein at 30 °C for 10 ns. RMSD (a) and number of H-bonds and salt bridges (b) were calculated for amino acid residues.

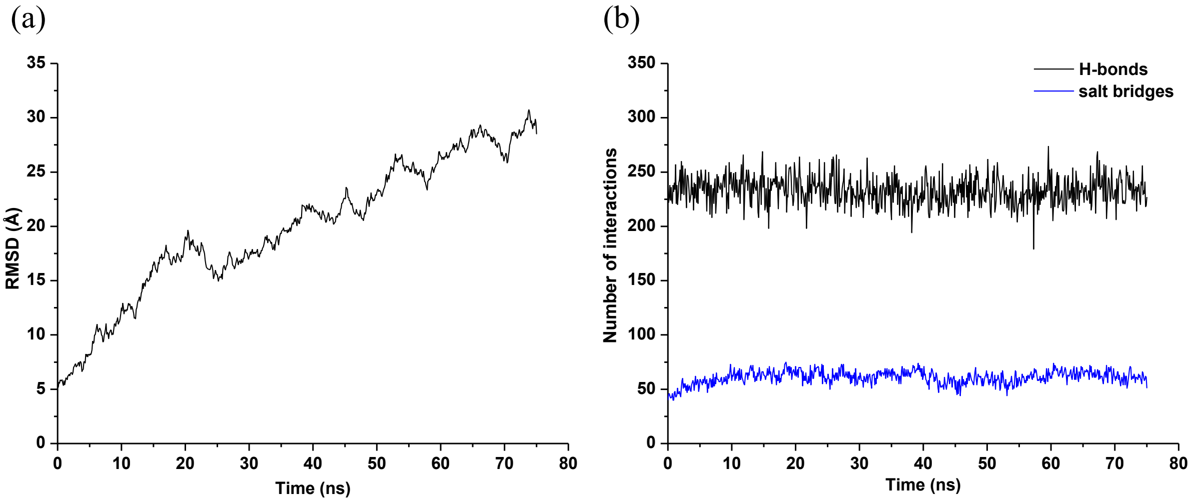

Figure S18. MD simulations of AGAM as a dimer and glycoprotein at 100 °C for 75 ns. RMSD (a) and number of H-bonds and salt bridges (b) were calculated for amino acid residues.

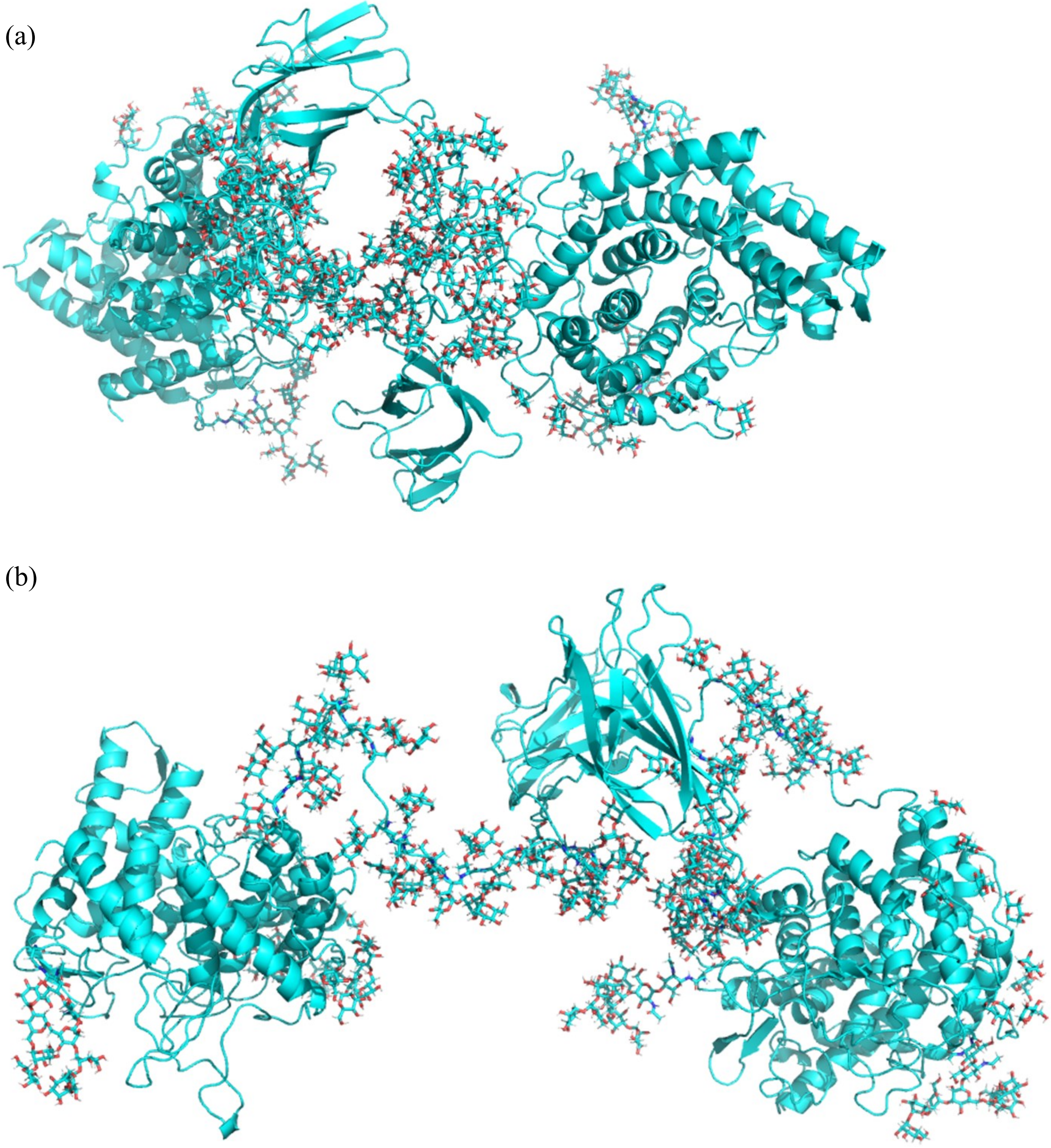

Figure S19. A dimer of AGAM obtained through MD simulations at 30 °C (a) and 100 °C (b). An initial model of the monomer of AGAM was created using AlphaFold 2.0, simulated at 30 °C for 75 ns, and aligned to form a dimer (a). A dimer was then simulated at 100 °C for 75 ns (b). *N*- and *O*-linked glycans are shown in sticks. *N*-linked glycans are $(Man)_8(GlcNAc)_2$ at glycosylation sites N170 and N394, while *O*-linked glycans are $(Man)_{1\text{-}2}$ in the linker sequence.
